# Tertiary lymphoid structure patterns aid in identification of tumor microenvironment infiltration and selection of therapeutic agents in bladder cancer

**DOI:** 10.3389/fimmu.2022.1049884

**Published:** 2022-11-07

**Authors:** Ye An, Jian-Xuan Sun, Meng-Yao Xu, Jin-Zhou Xu, Si-Yang Ma, Chen-Qian Liu, Zheng Liu, Shao-Gang Wang, Qi-Dong Xia

**Affiliations:** Department of Urology, Tongji Hospital, Tongji Medical College, Huazhong University of Science and Technology, Wuhan, Hubei, China

**Keywords:** tertiary lymphoid structures, tumor microenvironment, bladder cancer, immunotherapy, tumor mutation burden

## Abstract

**Background:**

Tertiary lymphoid structures (TLSs) are emerging as a potential predictor of prognosis and response to immunotherapy in some solid tumors. However, the comprehensive role of TLSs in bladder cancer remains unclear.

**Methods:**

Eighteen bladder cancer (BCa) datasets were downloaded from The Cancer Genome Atlas (TCGA), Gene Expression Omnibus (GEO), ArratyExpress and IMvigor210. Based on 39 validated TLS signature genes (TSGs), we evaluated the TLS patterns in all patients, and correlated the TLS patterns with prognosis and tumor microenvironment (TME) cell-infiltrating characteristics. The cox regression model and principal component analysis (PCA) algorithms were used to construct the TLS score, which helps to quantify the TLS pattern in individuals.

**Results:**

The landscape of 39 validated TSGs in BCa was assessed first. Five distinct TLS patterns and four gene clusters were determined. TLS cluster C2 and gene cluster A were thought to be characterized by mature TLSs and showed better prognosis and higher immune cells infiltration than other clusters. The TLS score was discovered to be tightly correlated with the infiltration level of immune cells, and could predict the maturation status of TLSs to some extent. We found TLS score was an excellent predictor for prognosis in patients with BCa independent of tumor mutation burden (TMB), and low TLS score was related to better prognosis than high TLS score. Besides, low TLS score was correlated with a better response to immune checkpoint blockade (ICB) immunotherapy and commonly used chemotherapy drugs.

**Conclusions:**

Our work demonstrated the characteristics of TLSs in BCa. By using the TLS score, we could evaluate the TLS pattern in individuals. Better understanding of TLS pattern and the usage of TLS score could help instruct clinical strategy and precision medicine for BCa.

## Introduction

Bladder cancer (BCa) is the tenth most common cancer worldwide with an estimated 573,000 new cases and 213,000 cancer deaths in 2020 (1). Based on the invasion of lamina propria, BCa can be divided into non-muscle invasive bladder cancer (NMIBC) and muscle invasive bladder cancer (MIBC). NMIBC represents approximately 70% of localized BCa and MIBC represents the remaining 30%. For prevention of recurrence or progression to MIBC, tumor resection followed by a scheduled intravesical instillation is the main treatment for NMIBC (2). Treatments for MIBC include neoadjuvant therapy followed by radical cystectomy (RC) and lymphadenectomy or a bladder-sparing project such as chemotherapy (3). With a more comprehensive understanding of tumor microenvironment (TME) and the rise of immune checkpoint blockade (ICB) therapy, immunotherapy offers a new option for patients with metastasized BCa (4). However, not all patients could benefit from immunotherapy. A single-arm phase 2 clinical trial with atezolizumab reported a 30% pathological complete response rate (5), thus, new biomarkers or molecular signatures are urgently needed to predict the efficacy of immunotherapy.

Tertiary lymphoid structures (TLSs) are ectopic lymphoid tissues formed at sites of long-lasting inflammation including tumors. Structurally resembling secondary lymphoid organs (SLOs), TLSs are mainly composed of B cells, T cells, dendritic cells (DCs), neutrophils and macrophages (6). TLSs also consist of high endothelial venules and lymphatic vessels, which help to guide the trafficking of immune cells into TME (7). Thus, TLSs play a nonnegligible role in anti-tumor immune activity, and it is reported that TLSs are correlated with better prognosis of most solid tumors including lung cancer (8, 9), breast cancer (10), colorectal cancer (11), pancreatic cancer (12) and melanoma (13). In addition, studies have revealed that B cells and TLSs are tightly associated with the response to immunotherapy (14), and the presence of TLSs is a predictive factor for the response to ICB therapy in sarcoma and melanoma (15, 16). Based on this, TLSs induction is now regarded as a potential therapeutic strategy for malignancies (17). Several studies have proved that widely used anti-cancer treatments could induce the formation of intratumoral TLSs in mouse models. For BCa, Zhou et al. and Pfannstiel et al. used public databases for bioinformatics analysis and found higher density of TLSs were correlated with a better response to ICB therapy and prognosis (18, 19).

However, the mechanisms behind the interaction between TLSs and BCa, and the crosstalk among immune cells in TLSs remain unclear. Meanwhile, the results of previous studies were based on the subtype of BCa, and none of them focused on the comprehensive effect of TLSs on BCa. Therefore, in this study, we integrated several independent BCa datasets and divided the patients with BCa into five TLS patterns according to TLS signature genes (TSGs). We performed survival analyses and investigated the landscape of TME cell infiltration in each pattern and found significantly different infiltration among five patterns. These were in accordance with the opinion that high heterogeneity existed in the cellular constituents of TLSs and would contribute to different anti-tumor effects and outcomes (14). Then, TLS cluster related differentially expressed genes (DEGs) were discovered, by which patients were classified into four genomic subgroups. The correlation between TLS patterns and gene patterns was evaluated. Finally, we developed a scoring system named TLS score to evaluate the TLS pattern in individuals and correlated it with tumor somatic mutation, TME cell infiltration characteristics, and response to immunotherapy and chemotherapy.

## Materials and methods

### Data retrieval and preprocessing

Thirty-nine TSGs were obtained from Fridman et al. (6). We selected data sets if they satisfied the following criteria. The inclusion criteria of the data sets are: 1) BCa patients with the results of transcriptome sequencing; 2) biological duplication should be more than 30; and the exclusion criteria is: normal people with transcriptome sequencing results. Transcriptional matrix (FPKM) and corresponding clinical information of 408 BCa patients was downloaded from The Cancer Genome Atlas (TCGA) (https://portal.gdc.cancer.gov/). Then, we transferred the fragments per kilobase million (FPKM) values into transcripts per kilobase million (TPM) values. EMTAB1803 and EMTAB4321 cohorts were downloaded from ArrayExpress ((https://www.ebi.ac.uk/arrayexpress). Transcriptional profiles and clinical information of 14 cohorts including GSE5287, GSE13507, GSE31684, GSE32548, GSE32894, GSE48075, GSE48276, GSE69795, GSE70691, GSE86411, GSE87304, GSE120736, GSE128192 and GSE128702 were downloaded from the Gene Expression Omnibus database (GEO, https://www.ncbi.nlm.nih.gov/gds). IMvigor210 immunotherapy cohort which investigated the therapeutic efficacy of Atezolizumab in metastatic urothelial carcinoma patients (20) was obtained from R software using R package “IMvigor210CoreBiologies”. The basic information of these included cohorts was shown in **Table 1**. Then we merged these 18 datasets and eliminated the batch effects using the Combat algorithm by R package “sva” (21). The principal components analysis (PCA) was used to check the effectiveness of the mergence. The copy number variation matrix was downloaded from UCSC-Xena (http://xena.ucsc.edu/).

**Table 1:** Data sources and including samples.

Data source	Tumor bulk-seq samples	Samples with survival data
E-MTAB-1803	85	73
E-MTAB-4321	476	0
GSE120736	145	0
GSE128192	112	0
GSE128702	256	0
GSE13507	188	164
GSE31684	93	93
GSE32548	131	0
GSE32894	308	0
GSE48075	142	73
GSE48276	116	73
GSE5287	30	30
GSE69795	61	38
GSE70691	49	49
GSE86411	132	0
GSE87304	305	0
IMvigor	348	348
TCGA	408	407
Meta-cohort	3385	1348

### The landscape of TSGs and TLS patterns in BCa

Systematic analyses of copy number variation and mutation of TSGs were conducted. Copy number variation and mutation analysis were visualized as mutation atlas and genome cycle plot respectively. Having merged the gene matrix and eliminated the batch effects, Kaplan–Meier method survival curve and log-rank test of each TSG were performed to investigate the prognostic value of TSGs.

Then, we conducted non-negative matrix factorization (NMF) algorithm based on the 39 TSGs to identify the TLS pattern. MCPCOUNTER and CIBERSORT methods (22) were applied to characterize the TME cell infiltration and quantify their proportion among TLS patterns. The differential overall survival was analyzed by log-rank test and Kaplan–Meier method survival curve to investigate survival benefits among these TLS patterns.

### Identification of DEGs among TLS patterns, DEGs based consensus cluster, and development of the TLS score

The DEGs were screened out by R package “limma” with the |log2 fold change (FC)| >0 and adjusted p-value <0.05, and finally visualized as an Upset diagram. Univariate Cox regression was performed to find out the DEGs with prognostic values. Subsequently, Gene Ontology (GO) and Kyoto Encyclopedia of Genes and Genomes (KEGG) enrichment analyses based on these DEGs were conducted. An unsupervised consensus cluster was performed by R package “ConsensusClusterPlus”. Similarly, the TME cell infiltration characteristics and the differential overall survival were analyzed among DEGs based consensus clusters. Then we distinguished the molecular characteristics of these DEGs with prognostic value by PCA algorithm and developed a TLS score formula: TLS score = ∑ (PC1 + PC2).

In this formula, PC1 and PC1 represent the expression levels of those DEGs with prognostic value in two different dimensions, respectively. TLS score was identified as the summary of PC1 and PC2, which can represent the individual TLS level to some degree.

### Validation of TLS score, and the correlation between TME and TLS score

Using the above formula, we calculated the TLS score of each sample. Following this, we checked the best cut-off value of TLS score in TCGA_BLCA cohort to gain the best prognostic predicting efficiency and obtained a threshold. All patients included in these 18 datasets were divided into low score group and high score group according to the threshold. Gene sets enrichment analysis (GSEA) was performed to find the differential function enrichments of TLS between high score group and low score group (23). We performed survival analyses in all patients and the above 18 cohorts separately to check whether the TLS score was a predictor of prognosis for BCa. Then, we used ssGSEA, ESTIMATE, TIMER, CIBERSORT, CIBERSORT-ABS, QUANTISEQ, MCPCOUNTER, XCELL and EPIC algorithms to investigate the correlation between TME and TLS score. Thus, we could obtain comprehensive characteristics of immune cells infiltration, immune related pathways and immune related functions between high and low groups. The correlation between TLS score and immune cells infiltration in the last seven algorithms was performed by the linear regression test. Besides, we calculated the tumor mutation burden (TMB) of each sample in TCGA_BLCA cohort and further investigated the correlation between TMB and TLS score. We also combined these two factors for prediction of overall survival in patients with BCa. Finally, we collected the mutation atlas of each sample and compared the differences in mutant frequencies between high and low TLS groups by the χ^2^ test.

### Prediction of response to chemotherapy/immunotherapy by TLS score

As TLSs were proved to be a predictor of response to ICB treatment in many solid tumors, we investigated the relationship between TLS score and drug sensitivity to either chemotherapy or immunotherapy. We used three algorithms to predict the response to immunotherapy: TCIA (24), TIDE (25) and SubMap (26). A novel algorithm called oncoPredict was used to predict the response to chemotherapy (27). All the prediction of response to chemotherapy or immunotherapy was compared between high TLS score group and low TLS score group by Wilcoxon or χ^2^ test.

### Statistical analysis

All the data processing, analyses and figure plotting were performed by R software vision 4.1.1. A P value less than 0.05 indicates statistical significance.

## Results

### The landscape of TSGs in BCa

**Figure 1:**
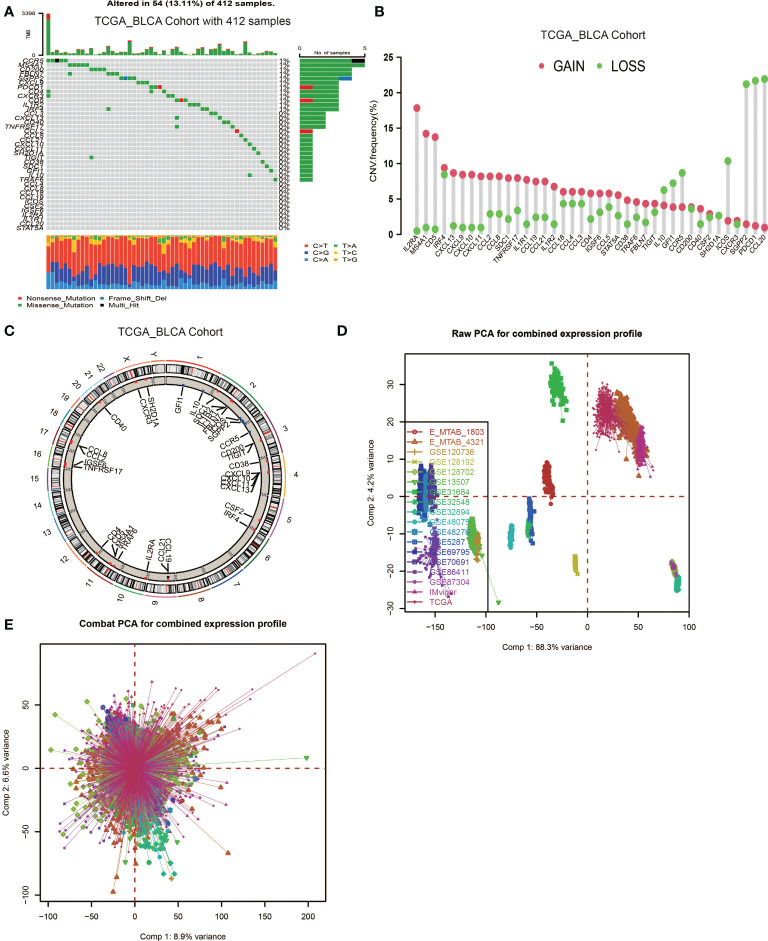
Landscape of genetic and expression variation of TLS signature genes in BCa and the combination of 18 datasets. **(A)** Mutation frequency of 39 TLS signature genes in 412 patients with BCa from the TCGA_BLCA cohort. Each column represented individual patients. The upper barplot showed TMB. The number on the right indicated the mutation frequency in each regulator gene. The right barplot showed the proportion of each variant type. The stacked barplot below showed a fraction of conversions in each sample. **(B)** CNV variation frequency of TLS signature genes. The height of the column represented the alteration frequency. The deletion frequency, green dot; The amplification frequency, red dot. **(C)** Location of CNV alteration of TLS signature genes on 23 chromosomes using cohort. **(D, E)** Principal component analysis for the expression profiles of common genes before and after combination of 18 cohorts. Before processing, 18 subgroups without intersection were identified, indicating these datasets samples were well distinguished based on the expression profiles of their common genes, while the 18 datasets merged well after processing.

In this study, 39 genes were identified as the gene signatures of TLSs, among which CCL2/3/4/5/8/18/19/21, CXCL9/10/11/13 were chemokine signature genes; CXCL13, CD200, FBLN7, ICOS, SGPP2, SH2D1A, TIGIT, PDCD1 were T follicular helper cell (T_FH_ cell) signature genes; CD4, CCR5, CXCR3, CSF2, IGSF6, IL2RA, CD38, CD40, CD5, MS4A1, SDC1, GFI1, IL1R1, IL1R2, IL10, CCL20, IRF4, TRAF6, STAT5A were T helper 1 cell (T_H_1 cell) and B cell signature genes; TNFRSF17 was plasma cell signature gene (6). We first explored the incidence of somatic mutations and copy number variations (CNV) of the 39 TSGs in BCa. These genes are immune-related, and we found low mutation rate (54 samples of 412 samples TCGA_BLCA cohort with a 13.11% frequency) in BCa (**Figure 1A**). Nevertheless, we found a prevalent alteration of CNV in all TSGs. Compared to the higher frequency of loss in IL10, GFI1, CCR5, ICOX, SGPP2, PDCD1 and CCL20, most TSGs had a greater frequency of CNV gain (**Figure 1B**). The locations of CNV were presented in **Figure 1C**.

Then, we used the Combat algorithm by R to eliminate the batch effects of the 18 included cohorts mentioned above and merged them into a new meta-cohort. Before processing, these datasets could easily be distinguished by principal component analysis (PCA) (**Figure 1D**), while they merged well after processing (**Figure 1E**). Following this, we divided the samples in the meta-cohort into two subgroups based on the expression level of each TSG and performed survival analyses. As shown in **Figures S2A–S**, higher expression level of CCL2, CCL8, CD4, CD5, CD38, CD40, CD200, CXCL9, CXCL10, CXCL13, CCR3, GFI1, ICOS, IRF4, MS4AA, PDCD1, SH2D1, STAT5 and TRAF6 showed a better survival advantage, while high expression level of IL1R1, IL10 and SDC1 indicated a worse prognosis (**Figures S1A–C**). CXCL13 was first described as a key chemokine for B cells migrating to SLOs, and was also regarded as a key regulator for TLSs formation. We found patients with high CXCL13 expression were associated with a significantly better prognosis (P < 0.001, **Figure S2J**).

### TLS patterns and the characteristics of TME cell infiltration

**Figure 2:**
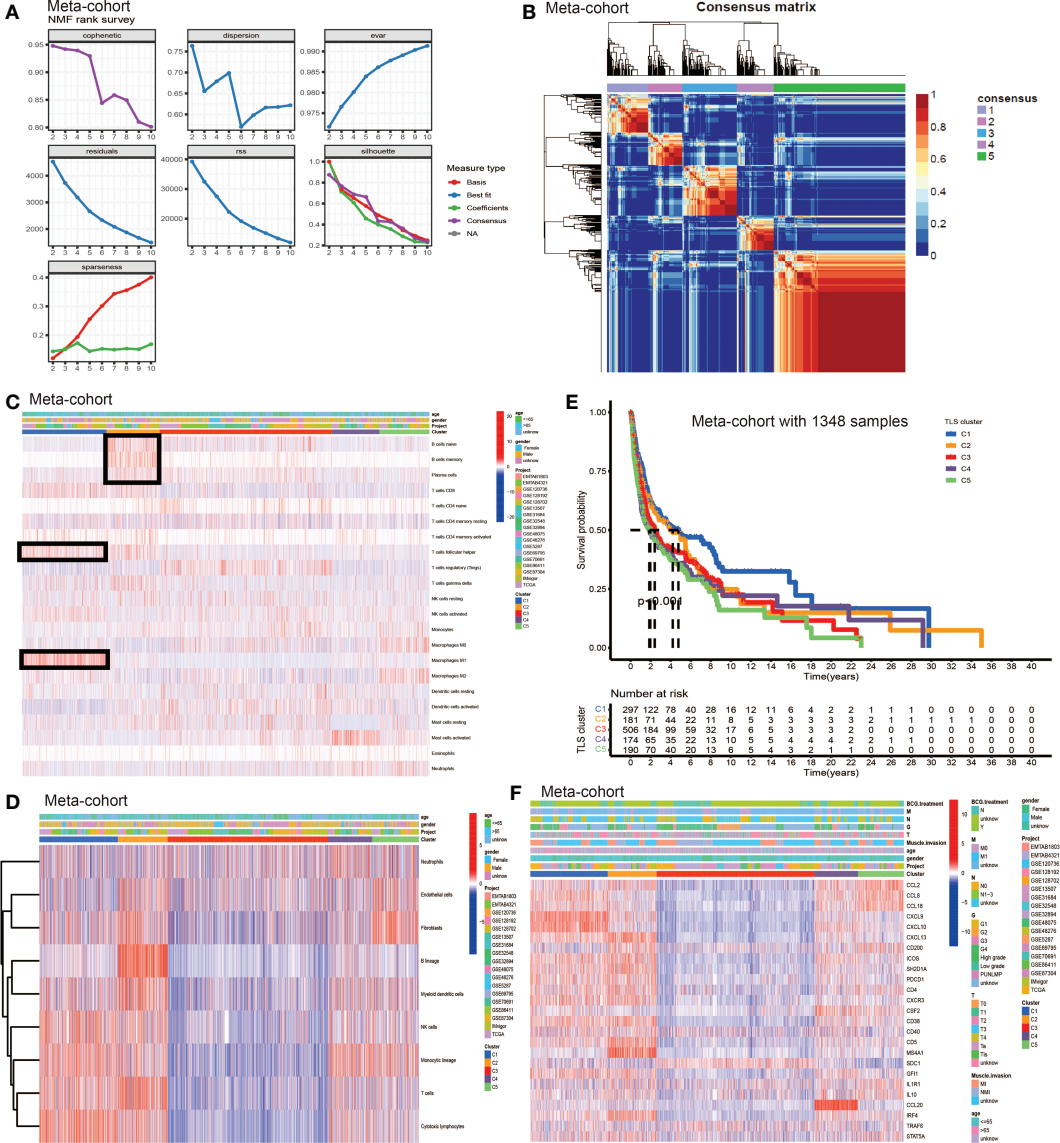
The generation of TLS patterns and biological characteristics of each pattern. **(A)** The NMF rank survey. **(B)** Connectivity matrix for patients with bladder cancer in the meta-cohort by NMF when k = 5. **(C)** TME cells infiltration characteristics in five different TLS patterns by CIBERSORT. **(D)** TME cells infiltration characteristics in five different TLS patterns by MCP. **(E)** Kaplan–Meier curves indicated TLS patterns were markedly related to overall survival of patients in meta-cohort. **(F)** TLS signature genes enrichment in each TLS pattern.

We used non-negative matrix factorization (NMF) algorithm for clustering, and could see an optimal clustering effect when k = 5 (**Figure 2A**). All samples in the meta-cohort were divided into 5 TLS patterns based on choosing k = 5, termed TLS cluster C1 – C5 (**Figure 2B**). We wondered whether there existed significant differences in immune cells infiltration among five TLS patterns. So we conducted TME infiltration analysis and discovered that TLS cluster C1 was significantly enriched in CD8^+^ T cell, T follicular helper cell (T_FH_ cell) and macrophage M1; TLS cluster C2 was significantly enriched in B cells (including naïve B cell, memory B cell and plasma B cell), CD8^+^ T cell, T_FH_ cell and myeloid dendritic cell (DC); TLS cluster C3 was enriched in regulatory T cell (T_reg_) while other immune cells showed low infiltration levels; cluster C4 showed enrichment in active mast cell and cluster C5 was enriched in macrophage M0, M2, fibroblasts, endothelial and neutrophil (**Figures 2C, D**). The TME cells infiltration characteristics were consistent with the results of survival analyses that patients from cluster C1 and C2 showed significant survival advantages compared to other clusters (**Figure 2E**). In addition, we investigated the TSGs signatures among five TLS patterns (**Figure 2F**), and we could see dramatic differences in 25 TSGs transcriptional profile among five clusters. Cluster C1 was characterized by the significantly increased expression level of CXCL9, CXCL10 and CXCL13; cluster C2 showed remarkable enrichment in CXCL13, ICOS, SH2D1A, CD4, CXCR3, CD38, CD5 and MS4A1; cluster C3 was enriched in SDC1, while other TGSs showed significantly decreased expression; cluster C4 was significantly enriched in CCL20; cluster C5 showed increased expression in CXCL2, CXCL8 and CXCL18 and decreased in SDC1 and CCL20.

### Generation of DEGs and the consensus clustering

**Figure 3:**
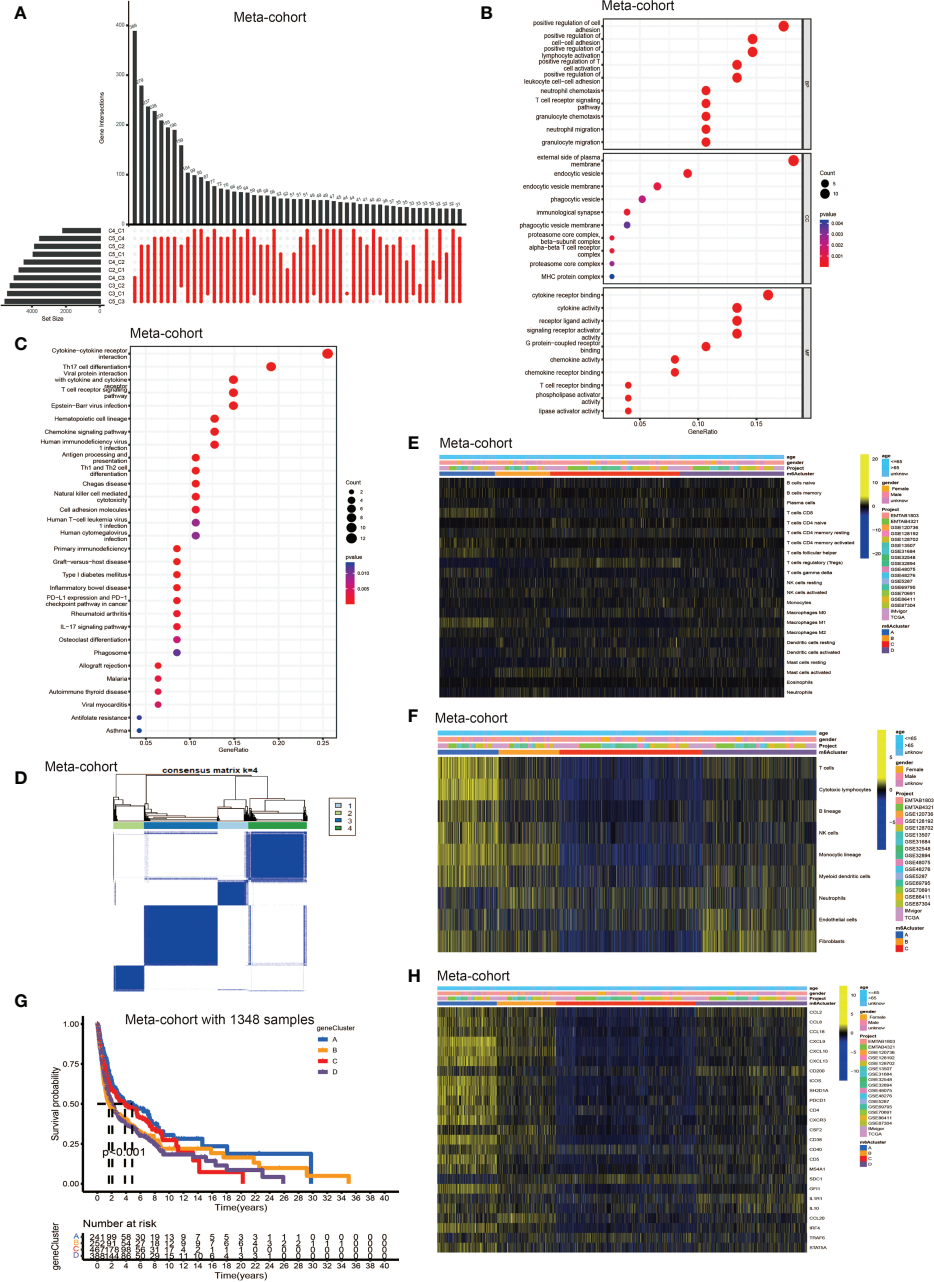
Identification of DEGs among TLS patterns and DEGs based consensus cluster. **(A)** 77 TLS cluster-related DEGs shown in the Upset diagram. **(B)** Functional annotation for TLS cluster related DEGs using GO enrichment analysis. The size of the plots represented the number of genes enriched. The pathways were grouped by cellular component (CC), molecular function (MF) and biological process (BP). **(C)** Functional annotation for TLS cluster related DEGs using KEGG enrichment analysis. The size of the plots represented the number of genes enriched. **(D)** Unsupervised clustering of 33 TLS cluster related DEGs with prognostic value in meta-cohort and consensus matrices for k = 4. **(E)** TME cells infiltration characteristics in four different TLS gene cluster by CIBERSORT. **(F)** TME cells infiltration characteristics in four different TLS gene cluster by MCP. **(G)** Kaplan–Meier curves indicated TLS genomic phenotypes were markedly related to overall survival of patients in meta-cohort. **(H)** TLS signature genes enrichment in each TLS gene cluster.

To further explore the latent mechanism behind the different characteristics among five TLS patterns, we found 77 TLS cluster related DEGs using R software package “limma” (**Figure 3A**). Cox regression model was used to screen out 33 DEGs with prognostic value. We first performed GO and KEGG enrichment analyses by clusterProfiler R package to find out the biological behavior behind these DEGs. We discovered enrichment in biological process (BP), namely, cell-cell adhesion, immune cell activation and granulocyte chemotaxis; cellular component (CC), plasma membrane, endocytic vesicle; molecular function (MF), namely, cytokine receptor binding, cytokine and chemokine activity (**Figure 3B**). The KEGG analysis showed similar results which exhibited high enrichment in cytokine-cytokine receptor reaction and T cell signaling pathway (**Figure 3C**). The above results proved again that TLSs were important in regulating and coordinating the complicated function of TME. Then, these 33 TLS related cluster DEGs were used for clustering analysis by the unsupervised clustering algorithm. By choosing k = 4 as the optimal k value, we finally divided patients into four genomic subgroups, named gene cluster A – D (**Figure 3D**, **Figures S3A–H**). Subsequently, we used the cumulative distribution function (CDF) curve to validate the rationality of grouping (**Figures S3I, J**), and the details of grouping were presented on the track plot (**Figure S3K**). Similarly, we investigated the differences in TME cell infiltration among these four gene clusters (**Figures 3E, F**). We discovered similar infiltration characteristics to TLS patterns that gene cluster A showed significantly high infiltration level of CD8^+^ T cell, B lineage, T_FH_, natural killer cell (NK cell) and macrophage M1; gene cluster B had high CD8^+^ T cell, NK cell, macrophage M1 and neutrophil infiltration; gene cluster C was enriched in T_reg_ while other immune cells showed significantly low level; gene cluster D was enriched in fibroblast, endothelial, and showed a relatively high level of macrophage M2 and B lineage. Survival analysis showed that patients from gene cluster A had the best prognosis than patients in cluster B - D (**Figure 3G**). Finally, we tested the expression level of TSGs among these five gene clusters (**Figure 3H**). Gene cluster A showed significantly high expression level of most TSGs except SDC1; cluster B exhibited relatively high expression of CCL8, CCL18, CXCL10, CXCL13, ICOS, SH2D1A, CSF2, CD38 and CCL20; cluster C only showed high SDC1 expression and had significantly decreased level of most TSGs; cluster D had high expression level of CCL2, CD200, IL1R1 and IL10.

### Development of TLS score and function annotation

**Figure 4:**
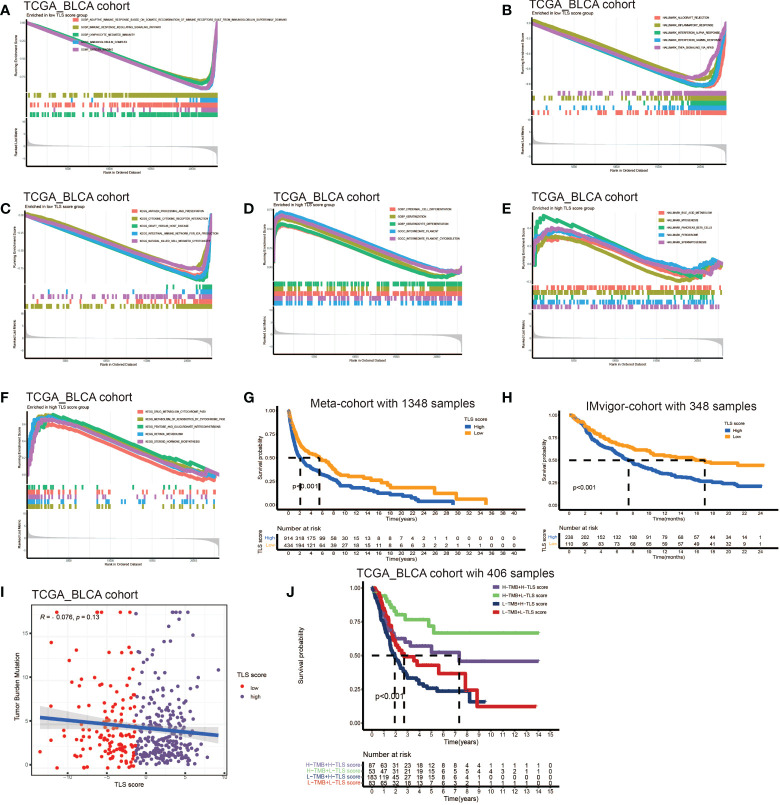
Development of TLS score and function annotation. **(A-C)** Gene sets enrichment analysis (GSEA) in low TLS score group by GO, KEGG and HALLMARK. **(D–F)** Gene sets enrichment analysis (GSEA) in high TLS score group by GO, KEGG and HALLMARK. **(G)** Survival analyses for low and high TLS score patient groups in meta-cohort using Kaplan–Meier curves (P <0.001, Log-rank test). **(H)** Survival analyses for low and high TLS score patient groups in IMvigor210 immunotherapy cohort using Kaplan–Meier curves (P <0.001, Log-rank test). **(I)** Linear regression analysis for tumor mutation burden and TLS score. The dot represented each sample. **(J)** Survival analyses for four groups grouped according to tumor mutation burden and TLS score in the TCGA_BLCA cohort using Kaplan–Meier curves.

The above analyses elucidated the landscape of TLS characteristics in BCa based on the patient population. However, the TLS patterns and gene clusters may not reveal the true situation of specific individuals due to the heterogeneity among patients. Therefore, we constructed a scoring system to quantify the TLS patterns in individuals. We named the scoring system TLS score, and we could classify patients into high TLS score group or low TLS score group based on this score. Firstly, we used gene sets enrichment analysis (GSEA) to investigate the differentially active pathways between the high and low groups. We discovered immune response, cytokines and antigen-antibody reaction pathways were significantly enriched in low TLS score group, such as adaptive immune response, immune response signaling pathway, lymphocyte mediated immunity, immunoglobulin complex, allograft pathway, inflammatory response, antigen processing, cytokine-receptor interaction (**Figures 4A–C**). While high TLS score group showed enrichment in metabolic related pathways and cell growth and differentiation related pathways, such as steroid hormone synthesis, retinol metabolism, drug metabolism cytochrome P450, and epidermal cell differentiation (**Figures 4D–F**). Then, we performed survival analysis in meta-cohort and the 18 cohorts separately (**Figures S4A–I**). Importantly, we observed a significant survival advantage in patients with low TLS score in the meta-cohort and IMvigor210 immunotherapy cohort (**Figures 4G, H**).

Tumor mutation burden (TMB) has been demonstrated to be a useful predictor for ICB treatment including BCa (28), and a pan-cancer research indicated that high somatic TMB was correlated with better prognosis of patients receiving ICB treatment than low TMB (29). Our result also revealed patients with high TMB had better prognosis compared to low TMB (**Figure S4A**). Subsequently, we analyzed the relationship between TLS score and TMB, and we didn’t find a significant correlation between them (**Figure 4I**). Therefore, we combined the TLS score and TMB to predict the prognosis (**Figure 4J**). We found patients with high TMB and low TLS score had the best overall survival and those with low TMB and high TLS had the worst. Additionally, we discovered patients with high TMB and low TLS score had better survival than high TMB and high TLS score. These analyses revealed TLS score was an excellent predictor of prognosis in patients with BCa independent of TMB, and had better effect than TMB at the same time. Finally, we investigated the distribution differences of somatic mutation between high score and low score groups in the TCGA_BLCA cohort. Generally, no obvious distribution differences were found between the two groups (**Figures S4B, C**), but significant differences existed in the distribution of gene FAT1, EPG5, AHNAK, ERBB2, PIK3CA, HERC1, RXRA, RNF213 and HYDIN (**Table 2**).

**Table 2:** Mutant genes that exist significant differences between high and low TLS score groups.

gene	H-wild	H-mutation	L-wild	L-mutation	p-value
FAT1	253(93.7%)	17(6.3%)	116(85.29%)	20(14.71%)	0.009425
EPG5	262(97.04%)	8(2.96%)	123(90.44%)	13(9.56%)	0.00946
AHNAK	254(94.07%)	16(5.93%)	117(86.03%)	19(13.97%)	0.011132
ERBB2	248(91.85%)	22(8.15%)	114(83.82%)	22(16.18%)	0.022194
PIK3CA	224(82.96%)	46(17.04%)	99(72.79%)	37(27.21%)	0.023353
HERC1	260(96.3%)	10(3.7%)	123(90.44%)	13(9.56%)	0.029162
RXRA	251(92.96%)	19(7.04%)	134(98.53%)	2(1.47%)	0.031326
RNF213	255(94.44%)	15(5.56%)	120(88.24%)	16(11.76%)	0.042807
HYDIN	259(95.93%)	11(4.07%)	123(90.44%)	13(9.56%)	0.046722

### TLS score and TME cell infiltration

**Figure 5:**
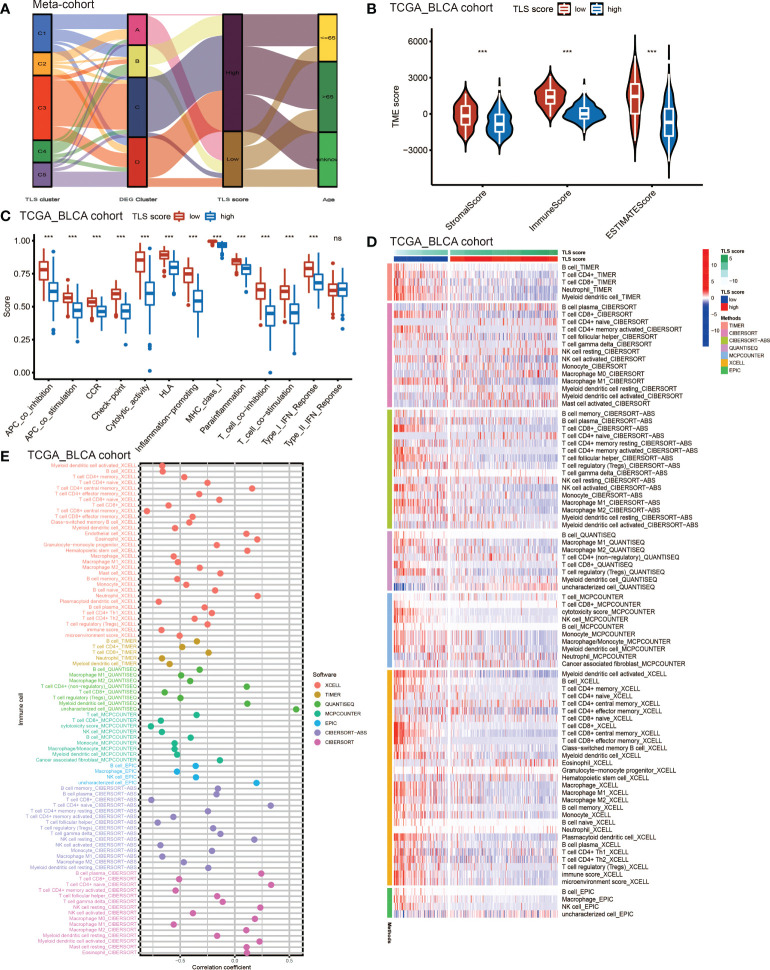
The TME cell infiltrating characteristics between high and low TLS score group. **(A)** Sankey diagram showing the changes of TLS clusters, gene clusters and TLS score and final survival status. **(B)** Differences in the stromal, immune and ESTIMATE score between high and low TLS score groups in meta-cohort (***P <0.001, Wilcoxon test). **(C)** The intensity of immune function between high and low TLS score groups. The upper and lower ends of the boxes represented interquartile range of values. The lines in the boxes represented median value, and black dots showed outliers. The asterisks represented the statistical p-value (ns, no significance; ***P < 0.001). **(D)** TME cells infiltration characteristics in high and low score groups by TIMER, CIBERSORT, CIBERSORT-ABS, QUANTISEQ, MCPCOUNTER, XCELL and EPIC methods. **(E)** The correlation between TLS score and infiltration level of immune cells.

**Figure 6:**
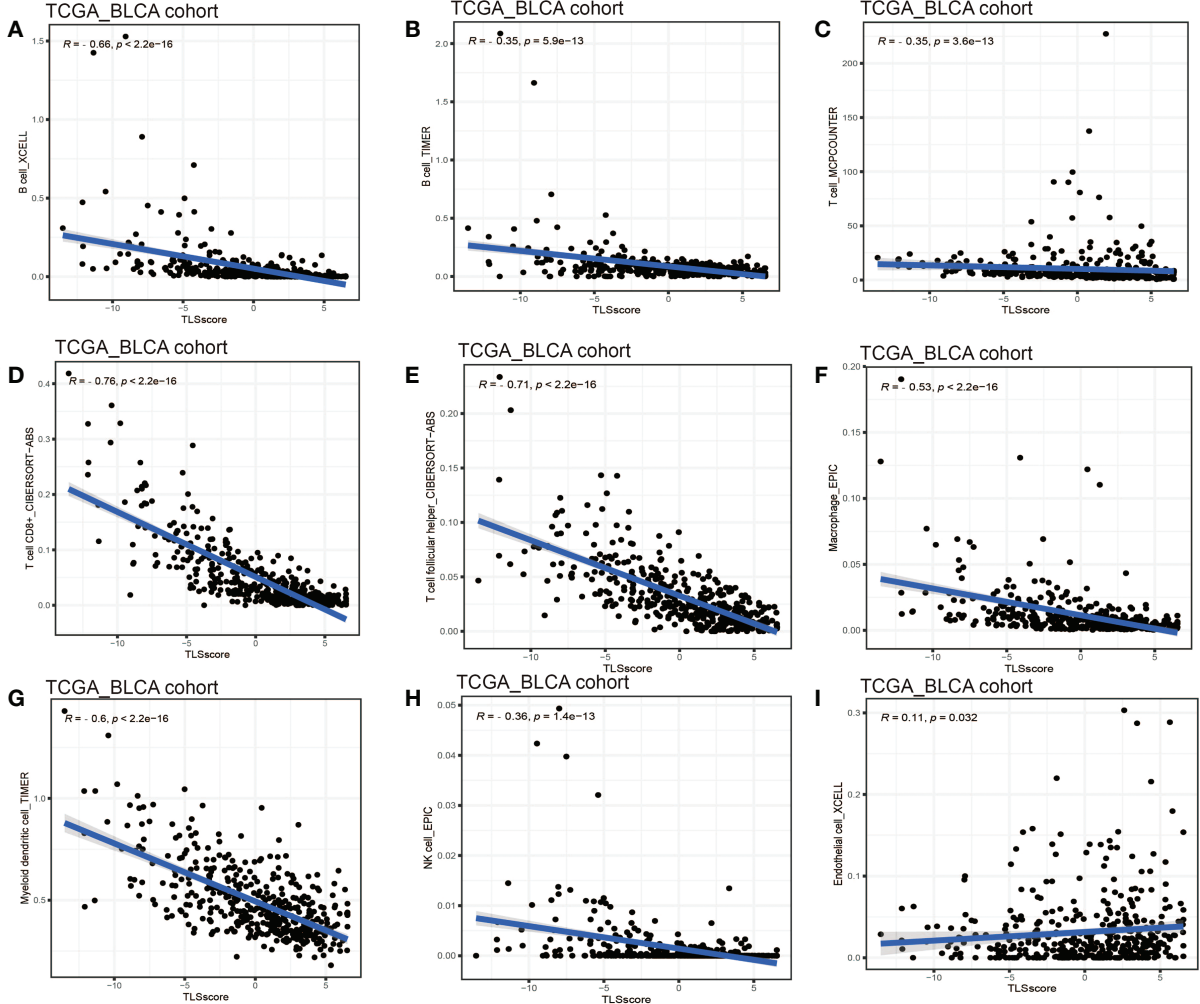
Linear regression analysis for TLS score and immune cells. **(A)** Correlation between TME B cells infiltration and TLS score by XCELL. **(B)** Correlation between TME B cells infiltration and TLS score by TIMER. **(C)** Correlation between TME T cells infiltration and TLS score. **(D)** Correlation between TME CD8^+^ T cell infiltration and TLS score. **(E)** Correlation between TME T cell follicular helper infiltration and TLS score. **(F)** Correlation between TME macrophages infiltration and TLS score. **(G)** Correlation between TME myeloid dendritic cell infiltration and TLS score. **(H)** Correlation between TME NK cell infiltration and TLS score. **(I)** Correlation between TME endothelial cell infiltration and TLS score.

Considering the crucial role of TLSs in anti-tumor immunity, we used nine algorithms to comprehensively investigate the characteristics of TME cell infiltration in two TLS score groups. The Sankey diagram showed the visualizing attribute changes in individual patients (**Figure 5A**). As shown in **Figure 5B**, low TLS score group was associated with higher immune score and ESTIMATE score. The ssGSEA showed the differences in immune function between two groups, and we could see low score group has better immune function in almost all the anti-tumor processes except IFN-β response (**Figure 5C**). TIMER, CIBERSORT, CIBERSORT-ABS, QUANTISEQ, MCPCOUNTER, XCELL and EPIC methods were used for the component analysis of TME cells (**Figure 5D**). We discovered low score group had significant high infiltration levels of B cells, T cells (CD4^+^ T cell and CD8^+^ T cell), macrophages (macrophage M1 and macrophage M2), myeloid dendritic cell and NK cell compared to high score group, and the lower of TLS score, the higher infiltration of immune cells. **Figure 5E** showed the correlation between TLS score and infiltration level of immune cells. We noticed the value of TLS score showed significantly negative correlation with B cells (**Figures 6A, B**) including naïve B cells, memory B cells and plasma cells (**Figures S5A–E**); T cells including various types of CD4^+^ T cell, CD8^+^ T cell and T_FH_ cell (**Figures 6C–E** and **Figure S5F–K**); macrophages including macrophage M1 and macrophage M2 (**Figure 6F** and **Figures S5L, M**). Besides, TLS score had significantly negative correlation to myeloid dendritic cells and NK cells (**Figures 6G, H**). We also noticed that TLS score was positively correlated with endothelial cells (**Figure 6I**). In general, the TLS score was significantly associated negatively with the infiltration level of most immune cells.

### Characteristics of TLS in immunotherapy and chemotherapy

**Figure 7:**
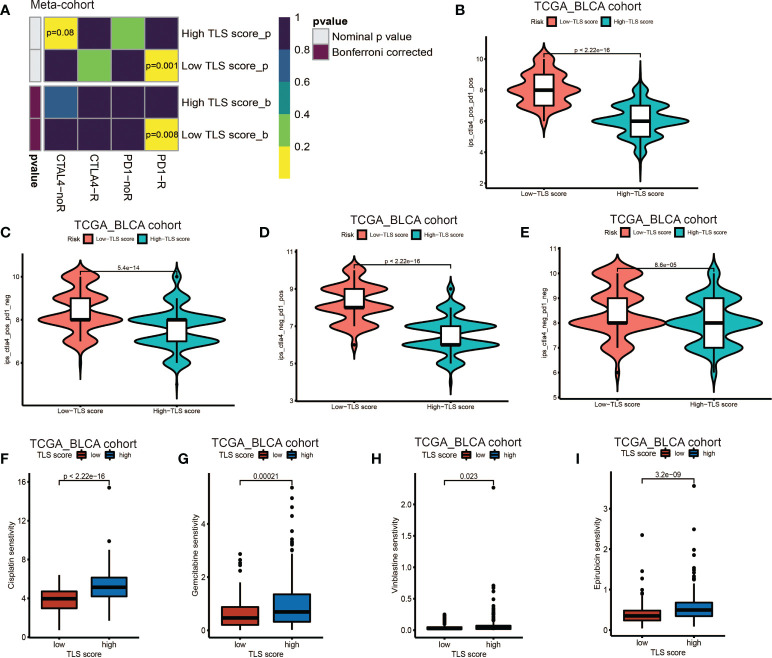
Role of TLS patterns in immunotherapy and chemotherapy. **(A)** The similarity of gene expression profiles between TLS score and bladder cancer patients treated with immune checkpoint blockade (ICB). CTLA4-noR, patients no respond to anti-CTLA4 treatment, CTLA4-R, patients respond to anti-CTLA4 treatment, PD1-noR, patients no respond to anti-PD-1 treatment, PD1-R, patients respond to anti-PD-1 treatment. **(B–E)** The violin diagram showed the differences of response index between high and low TLS score groups among four subgroups. **(F–I)** The differences of drug sensitivity (oncoPredict score) between high and low TLS score group. **(F)** Cisplatin for chemotherapy; **(G)** Gemcitabine for chemotherapy; **(H)** Vinblastine for chemotherapy; **(I)** Epirubicin for intravesical instillation.

Our above results demonstrated the TLS score was an excellent predictor for prognosis and was tightly correlated with infiltration level of immune cells. Based on this, we proposed that low TLS score might indicate an immune subtype which was more sensitive to immunotherapy. Therefore, we investigated the correlation between the TLS score and the response to ICB treatment. We discovered low TLS score was significantly associated with better response to anti-PD-1 immunotherapy and the significant correlation still existed after Bonferroni correction (**Figure 7A**). Then, the patients were classified into four subgroups according to their usage of anti-PD-L1 and anti-CTLA-4 treatments: CTLA-4 positive PD-1 positive (**Figure 7B**), CTLA-4 positive PD-1 negative (**Figure 7C**), CTLA-4 negative PD-1 positive (**Figure 7D**), CTLA-4 negative PD-1 negative (**Figure 7E**). We found that in all four subgroups, the low TLS score group had significant higher IPS score than low TLS score group, which indicated patients from low TLS score group were associated with better response to anti-PD-1, anti-CTLA-4 or combined immunotherapy compared to high TLS score. We also performed Tumor Immune Dysfunction and Exclusion (TIDE) analysis, and surprisingly found low TLS score was related to higher TIDE score (**Figure S6A**). This immune evasion effect might be caused by the relatively higher infiltration level of T_reg_ in low TLS score group (**Figure 4C**).

Additionally, we investigated the relationship between TLS score and response to chemotherapy. We applied R package oncoPredict and examined several commonly used drugs, such as gemcitabine, cisplatin and vinblastine which were used for adjuvant treatment after surgery and epirubicin which was used for intravesical instillation. We found low TLS score was significantly related to lower sensitivity score of gemcitabine, cisplatin, vinblastine and epirubicin (**Figures 7F–I**), which indicated that low TLS score group was associated with higher sensitivity to chemotherapy. Several other chemotherapy drugs were also screened out, and showed significant association with TLS score (**Figure S6B–X** and **Figures S7A–Y**). Although some of them are newly developed and haven’t been used for BCa treatment, we could screen out appropriate chemotherapy drugs for patients and instruct clinical medication in the future.

## Discussion

TLSs have emerged as a crucial role in the immune response of anti-tumor effect and at the same time as a predictor of prognosis and response to immunotherapy. As mentioned earlier, many researches have been carried out to explore the complex role of TLSs in anti-cancer, but there are still many mysteries. For example, there still lacks accurate and comprehensive biomarkers of TLSs, although many biomarkers have been continuously proposed (17). Meanwhile, the mechanisms how TLSs regulate the immune response and how the immune cells interact with each other (especially B cells and T cells) also remain unclear (30). Therefore, it is necessary and urgent to investigate the comprehensive role of TLSs in malignancies. Although previous studies have explored the predictive value of TLSs in BCa, their results were based on either the immune subtype or limited TLSs signature biomarkers, which may lead to a biased conclusion. For example, Zhou et al. divided patients with MIBC into six MIBC immune classes, and found class F had the best prognosis and highest level of TLSs. In addition, they analyzed the TLS signature in pan-cancer using nine genes validated in metastasized melanoma tumors as TLSs signature genes (19). Pfannstiel et al. quantified the TLSs in intra- and peritumoral stroma, and found the number of TLS and the distance from TLS to tumor were associated with the disease specific survival. These studies paid more attention to the TME of BCa or limited TLSs signature genes and didn’t go deep into the effects of TLSs in BCa.

In our study, we enrolled 18 bladder cancer datasets and 39 validated TSGs for further investigation which was the most comprehensive analysis of the role of TLSs in BCa currently reported. Firstly, we summarized the landscape of the 39 validated TSGs in BCa and discovered most TSGs exhibited CNV rather than somatic mutation. Most TSGs including the essential chemokines for the formation of TLSs such as CXCL13 and CCL21 showed greater frequency of CNV gain, which indicated the neogenesis of TLSs in tumor tissue. Recently, Groeneveld et al. proved CXCL13 could also be regarded as the biomarker of TLSs in BCa, and demonstrated CXCL13 was associated with better prognosis of patients with BCa (31). Similarly, CCL21 is crucial for the recruitment of lymphocytes and TLSs formation. Delvecchio et al. elucidated that intratumoral injection of CXCL13 and CCL21 could induce TLSs formation in the orthotopic model of pancreatic tumor, resulting in a better therapeutic effect of gemcitabine (32). Then, we merged the 18 cohorts into a new meta-cohort, and used the NMF clustering to divide all patients into five TLS patterns named TLS pattern C1 – C5 according to the expression level of TSGs. It’s not surprising to find there existed huge differences between five TLS patterns among the various aspects from TME cell infiltration to prognosis. Cluster C2 showed high infiltration level of B cells (including naïve B cell, memory B cell and plasma B cell), CD8^+^ T cell, T_FH_ cell and myeloid dendritic cell. It is now considered that TLSs could be divided into three different mature states: early TLS, composed of dense lymphocytic aggregate but lacking DCs; immature TLS, having DCs but lacking germinal center (GC); and mature TLS, having active GC and active B cell (33–35). Therefore, we thought cluster C2 was characterized by mature TLS, and not surprising to find patients from cluster C2 had the best survival advantage. Cluster C1 was significantly enriched in CD8^+^ T cell, T_FH_ cell and macrophage M1. T_FH_ (CD4^+^ CXCL13^+^ T cell) which could produce CXCL13 is also believed crucial in the formation of TLSs (6, 36). Although we didn’t see significantly high infiltration level of B cells, cluster C1 showed better prognosis than clusters C3 – C5. We supposed that the survival advantage was due to the significantly high infiltration of T_FH_ which indicated active TLS neogenesis. The following heatmap of TGSs also proved our opinion. Notably, CCL20 was highly enriched in cluster C4 and we observed patients from cluster C4 had the worst survival advantage. Previous studies have proved that the activation of CCL20-CCR6 axis could promote ovarian cancer migration, lung adenocarcinoma progression and impair the function of T cells in prostate cancer (37–39). Thus, our findings could provide a new insight into CCL20 in BCa.

Next, we investigated the mRNA transcriptome differences between distinct TLS patterns and used unsupervised clustering to divide patients into four gene clusters named gene cluster A – D. Consistent with the TLS clusters, gene cluster A was characterized by mature TLS and patients from it had the best prognosis. Interestingly, we found gene clusters B and D had relatively high infiltration levels of immune cells (such as B cells, T cells, macrophages and neutrophils), but didn’t show corresponding survival advantages. Thus, we thought gene clusters B and D were characterized by immune-exclude phenotype, also called ‘cold’ tumor (40). In this phenotype, cytotoxic T lymphocytes (CTLs) were excluded from the core of the tumor and instead present along the margin of the tumor where they could be stuck in the fibrotic stroma. The significantly high infiltration level of fibroblasts and endothelial cells in clusters B and D also proved it.

To further investigate the TLS pattern in individuals, we developed a scoring system named TLS score to quantify the TLS pattern in patients with BCa. Using the TLS score, we divided all patients into two subgroups: high TLS score group and low TLS score group. We first performed GSEA to dig out the latent enrichment of pathways between two subgroups. We found immune response, cytokines and antigen-antibody reaction related pathways were enriched in low score group, while metabolic related pathways and cell growth and differentiation related pathways were enriched in high score group. This result revealed a better anti-tumor potential of low score group compared to high score group. The subsequent survival analysis also proved it that patients from low score group had significantly better prognosis than that in high score group. As TMB has been proved as a biomarker for prediction of response to ICB treatment (29), we further evaluated the relationship between TLS score and TMB. We didn’t find significant correlation between TLS score and TMB, which indicated TLS score might be a predictor independent of TMB. So, we combined TMB and TLS score for prognosis prediction. Consistent with our expectations, TLS score showed a better predictive value of prognosis than TMB. Nine genes (FAT1, EPG5, AHNAK, ERBB2, PIK3CA, HERC1, RXRA, RNF213 and HYDIN) showed significant distribution differences of somatic mutation between high and low score groups. Among them, five genes have been reported to be correlated with the development, progression or metastasis of BCa. For example, Wang et al. reported that knockdown of FAT1 promoted the BCa cell apoptosis and inhibit the viability and migration of BCa *in vitro* (41). The overexpression of AHNAK has been reported to promote the proliferation and migration of UMUC3 and T24 cells, while knockdown of AHNAK could inhibit the metabolism and epithelial-mesenchymal transition (EMT) of these cells (42). Besides, RXRA hot-spot mutant was reported to related to upregulated PPAR signaling activity (43), and then contributed to the growth of BCa cells (44). The other genes (EPG5, HERC1, RNF213 and HYDIN) haven’t been reported to correlate with the pathology of BCa, and we think these genes are potential targets for intervention of BCa.

A systematic analysis of immune cell infiltration in the TME was conducted. We found low TLS score group showed better immune functions than high score group and obtained higher ESTIMATE score. Component analysis of infiltrating cells revealed that low score group had significantly high infiltration level of most immune cells: B cells, T cells (CD4^+^ T cell and CD8^+^ T cell), macrophages (macrophage M1 and macrophage M2), myeloid dendritic cell and NK cell. However, we noticed T_reg_ cell also showed infiltration in low TLS score group. T_reg_ cell is an important immunosuppressive cell and usually leads to tumor angiogenesis, immune evasion, drug resistance, tumor progression and metastasis (45). Indeed, T_reg_ cell was found within TLSs in breast cancer, lung cancer, colorectal cancer and prostate cancer, and contributed to a negative effect on the capacity of TLSs (46–48). The research results of Joshi et al. demonstrated T_reg_ cells suppressed anti-tumor response in TLSs, while after T_reg_ cell depletion, T cells proliferation rates increased in TLSs and led to tumor destruction (49). These results suggest TLSs play a complicated role in anti-tumor effect, and more researches and accurate biomarkers for TLSs are needed in the future. Correlation analysis of infiltrated immune cells showed the value of TLS score was negatively correlated with B cells (naïve B cell, memory B cell and plasma cell), T cells (CD4^+^ T cell, CD8^+^ T cell and T_FH_ cell), macrophages (macrophage M1 and M2), myeloid dendritic cell and NK cell. Therefore, the TLS score could be used to evaluate the infiltration level of immune cells in TME in individual patients with BCa. Considering plasma cells in TLSs were activated and differentiated in GC, the TLS score could also reveal the maturation state of TLSs to some extent. In recent years, humoral immunity has been proposed to be important in anti-tumor effects, and B cells play a key role in this process. A pan-cancer study demonstrated that the presence of memory B cells was associated with poor prognosis in colon, gastric cancers, although TLSs were reported to promote the prognosis of patients in these cancers (50). Another study reported that high density of B cells was related to better prognosis of patients with pancreatic cancer but only if these cells formed TLS (51). Therefore, when considering the anti-tumor effects of B cells, it is important to distinguish the phenotypes or characteristics of B cells. For BCa, Koti et al. reported that well-formed TLS were more common in aggressive high grade MIBC compared to low grade NIMBC (52). More studies are needed to further elucidate the effects of B cells in BCa.

Immunotherapy, led by ICB treatment (PD-1/PD-L1 blockade alone or combined with CTLA-4 checkpoint inhibition) showed great benefit in the second-line therapy of patients with unresectable and metastatic BCa (53). However, the response rate to ICB treatment is low, and there is an urgent need to find new biomarkers to screen out patients who are appropriate for ICB treatment. Previous studies have reported the presence of TLSs was associated with higher response rate to immunotherapy of ICB treatment in the patient population with BCa. Here we wondered whether TLS score could predict the response to ICB treatment in individuals and instruct the clinical treatment strategy. We found low TLS score group was significantly associated with the response to anti-PD-1 treatment. In TCIA method, we discovered patients from low score group showed advantages not only in anti-PD-1/anti-CTLA-4 monotherapy but in combined immunotherapy. Besides, survival analysis revealed that patients from low score group had better prognosis than that from high score group in meta-cohort and IMvigor210 immunotherapy cohort. These results demonstrated that TLS score was an excellent predictor for the response to ICB immunotherapy and prognosis in patients with BCa. To our surprise, in TIDE analysis, low TLS score group had a relatively higher TIDE score than high TLS score group. We thought the result of TIDE might be influenced by the relatively high T_reg_ cell infiltration level and led to bias.

Finally, we investigated the relationship between TLS score and response to chemotherapy by oncoPredict algorithm. For BCa, gemcitabine, cisplatin and vinblastine are usually used for adjuvant treatment after surgery and epirubicin is usually used for intravesical instillation. We found low TLS score group was related to better response to all these drugs. In addition, we screen out a number of other chemotherapy drugs which showed better response in low TLS score group. Our findings revealed that TLS score played a unique role in predicting the response to chemotherapy in BCa. Exhilaratingly, previous studies have reported that widely used anti-cancer drugs could induce the formation of intratumoral TLSs in mice. In patients with cancer, studies revealed the use of chemotherapy was associated with massive TLSs in tumors (54, 55), which strongly indicated that chemotherapy could induce the formation of TLSs. All these results suggest TLSs and chemotherapy are important in tumor destruction, and mutually reinforce anti-tumor effect. Our work plays a key role in this process that help to screen out patients who are appropriate for chemotherapy.

In general, we used the validated TGSs to provide a comprehensive insight into TLSs in BCa and evaluated the comprehensive role of TLSs in prognosis, TMB, TME immune cell infiltration, response to chemotherapy and immunotherapy. The TLS patterns could help to distinguish patients with different statuses of TLSs, and draw the landscape of TME cell infiltration, TSGs expression and prognosis among patients. The TLS score could evaluate the specific TLS pattern in individuals, and was proven to be a good predictor for prognosis, response to immunotherapy and chemotherapy. Besides, TLS score showed a significant correlation with the infiltration level of immune cells and could indicate the maturation status of TLSs to some extent. Therefore, the TLS score could aid in precision medicine for patients with BCa.

However, there are a few limitations existing in our study. First, though 39 genes were validated as TSGs, there still lacks accurate TSGs. The TSGs we included in this study might not be comprehensive and accurate enough, thus, might lead to bias. Second, the study was performed by bioinformatic analyses, and many cells reported to play a role in TLSs recently such as regulatory B (B_reg_) cells couldn’t be distinguished well by algorithms. Third, although the analysis used data from 18 cohorts and clinical samples size is relatively adequate, our study lacks external verification in clinical trials. Finally, the exact mechanisms behind the interaction between TLSs and BCa remain unclear, and more researches are needed to further unveil the mystery of TLSs.

In conclusion, our work demonstrated the characteristics of TLSs in BCa. By using the TLS score, we could evaluate the TLS pattern in individuals, and predict the TME cell infiltration, TLS maturation, prognosis, response to immunotherapy and chemotherapy in BCa. Thus, better understanding of TLS pattern and the usage of TLS score could help instruct clinical strategy and improve prognosis of patients with BCa.

## Data availability statement

The datasets presented in this study can be found in online repositories. The names of the repository/repositories and accession number(s) can be found in the article/**Supplementary Material**.

## Author contributions

Q-DX, S-GW and ZL designed the study. Q-DX collected the data, analyzed the data and drew the figures. YA and J-XS analyzed the data and wrote the manuscript. M-YX, C-QL, J-ZX and S-YM contributed to critical revision of the manuscript. All authors contributed to the article and approved the submitted version.

## Funding

This work was supported by the National Natural Science Foundation of China (81772729) and Undergraduate Training Program for Innovation and Entrepreneurship (S202210487014).

## Acknowledgments

We thank all the R software package developers.

## Conflict of interest

The authors declare that the research was conducted in the absence of any commercial or financial relationships that could be construed as a potential conflict of interest.

## Publisher’s note

All claims expressed in this article are solely those of the authors and do not necessarily represent those of their affiliated organizations, or those of the publisher, the editors and the reviewers. Any product that may be evaluated in this article, or claim that may be made by its manufacturer, is not guaranteed or endorsed by the publisher.

## Supplementary material

Supplementary Figure 1 Survival analysis for TLS signature genes that low expression subgroup showed survival advantages. **(A)** Survival analysis for IL1R1. **(B)** Survival analysis for IL10. **(C)** Survival analysis for SDC1.

Supplementary Figure 2 Survival analysis for TLS signature genes that high expression subgroup showed survival advantages. (**A–S)** Survival analysis for CCL2, CCL8, CD4, CD5, CD38, CD40, CD200, CXCL9, CXCL10, CXCL13, CCR3, GFI1, ICOS, IRF4, MS4AA, PDCD1, SH2D1, STAT5 and TRAF6.

Supplementary Figure 3 The process of generating four distinct TLS cluster related DEGs genomic patterns. **(A–H)** Unsupervised clustering of 33 TLS cluster related DEGs in meta-cohort and consensus matrices for k = 1, 3 - 9. **(I)** The cumulative distribution function (CDF) curve for k = 2 - 9. **(J)** The scree plot for k = 2 - 9. K The track plot for k = 2 - 9.

Supplementary Figure 4 Survival analysis between high and low TMB subgroups and somatic mutation between high and low TLS score groups. A Survival analysis between high and low TMB subgroups. B Distribution differences of somatic mutation between high score and low score groups in the TCGA_BLCA cohort.

Supplementary Figure 5 Linear regression analysis for TLS score and immune cells. **(A)** B cell memory. **(B)** B cell memory. **(C)** B cell naive. **(D)** B cell plasma. **(E)** B cell plasma. **(F)** T cell CD4^+^ Th1. **(G)** T cell CD4^+^ Th2. **(H)** T cell CD4^+^ memory actived. **(I)** T cell CD8^+^. **(J)** T cell CD8^+^ effector memory. **(K)** T cell follicular helper. **(L)** Macrophage M1. **(M)** Macrophage M2.

Supplementary Figure 6 23 drugs that are correlated with TLS score.

Supplementary Figure 7 25 drugs that are correlated with TLS score.

## References

[1] Sung H Ferlay J Siegel RL Laversanne M Soerjomataram I Jemal A . Global cancer statistics 2020: GLOBOCAN estimates of incidence and mortality worldwide for 36 cancers in 185 countries. CA Cancer J Clin (2021) 71(3):209–49. doi: 10.3322/caac.21660 33538338

[2] Babjuk M Burger M Capoun O Cohen D Compérat EM Dominguez Escrig JL . European Association of urology guidelines on non-muscle-invasive bladder cancer (Ta, T1, and carcinoma in situ). Eur Urol (2022) 81(1):75–94. doi: 10.1016/j.eururo.2021.08.010 34511303

[3] Lenis AT Lec PM Chamie K Mshs MD . Bladder cancer: A review. Jama (2020) 324(19):1980–91. doi: 10.1001/jama.2020.17598 33201207

[4] Wu Z Liu J Dai R Wu S . Current status and future perspectives of immunotherapy in bladder cancer treatment. Sci China Life Sci (2021) 64(4):512–33. doi: 10.1007/s11427-020-1768-y 32926318

[5] Powles T Kockx M Rodriguez-Vida A Duran I Crabb SJ van der Heijden MS . Clinical efficacy and biomarker analysis of neoadjuvant atezolizumab in operable urothelial carcinoma in the ABACUS trial. Nat Med (2019) 25(11):1706–14. doi: 10.1038/s41591-019-0628-7 31686036

[6] Sautès-Fridman C Petitprez F Calderaro J Fridman WH . Tertiary lymphoid structures in the era of cancer immunotherapy. Nat Rev Cancer (2019) 19(6):307–25. doi: 10.1038/s41568-019-0144-6 31092904

[7] Ruddle NH . High endothelial venules and lymphatic vessels in tertiary lymphoid organs: Characteristics, functions, and regulation. Front Immunol (2016) 7:491. doi: 10.3389/fimmu.2016.00491 27881983 PMC5101196

[8] Dieu-Nosjean MC Antoine M Danel C Heudes D Wislez M Poulot V . Long-term survival for patients with non-small-cell lung cancer with intratumoral lymphoid structures. J Clin Oncol (2008) 26(27):4410–7. doi: 10.1200/JCO.2007.15.0284 18802153

[9] Germain C Gnjatic S Tamzalit F Knockaert S Remark R Goc J . Presence of b cells in tertiary lymphoid structures is associated with a protective immunity in patients with lung cancer. Am J Respir Crit Care Med (2014) 189(7):832–44. doi: 10.1164/rccm.201309-1611OC 24484236

[10] Martinet L Garrido I Filleron T Le Guellec S Bellard E Fournie JJ . Human solid tumors contain high endothelial venules: association with T- and b-lymphocyte infiltration and favorable prognosis in breast cancer. Cancer Res (2011) 71(17):5678–87. doi: 10.1158/0008-5472.CAN-11-0431 21846823

[11] Di Caro G Bergomas F Grizzi F Doni A Bianchi P Malesci A . Occurrence of tertiary lymphoid tissue is associated with T-cell infiltration and predicts better prognosis in early-stage colorectal cancers. Clin Cancer Res (2014) 20(8):2147–58. doi: 10.1158/1078-0432.CCR-13-2590 24523438

[12] Hiraoka N Ino Y Yamazaki-Itoh R Kanai Y Kosuge T Shimada K . Intratumoral tertiary lymphoid organ is a favourable prognosticator in patients with pancreatic cancer. Br J Cancer (2015) 112(11):1782–90. doi: 10.1038/bjc.2015.145 PMC4647237 25942397

[13] Ladányi A Kiss J Somlai B Gilde K Fejos Z Mohos A . Density of DC-LAMP(+) mature dendritic cells in combination with activated T lymphocytes infiltrating primary cutaneous melanoma is a strong independent prognostic factor. Cancer Immunol Immunother CII. (2007) 56(9):1459–69. doi: 10.1007/s00262-007-0286-3 PMC11030123 17279413

[14] Helmink BA Reddy SM Gao J Zhang S Basar R Thakur R . B cells and tertiary lymphoid structures promote immunotherapy response. Nature (2020) 577(7791):549–55. doi: 10.1038/s41586-019-1922-8 PMC8762581 31942075

[15] Petitprez F de Reyniès A Keung EZ Chen TW Sun CM Calderaro J . B cells are associated with survival and immunotherapy response in sarcoma. Nature (2020) 577(7791):556–60. doi: 10.1038/s41586-019-1906-8 31942077

[16] Cabrita R Lauss M Sanna A Donia M Skaarup Larsen M Mitra S . Tertiary lymphoid structures improve immunotherapy and survival in melanoma. NatureS (2020) 577(7791):561–5. doi: 10.1038/s41586-019-1914-8 31942071

[17] Fridman WH Meylan M Petitprez F Sun CM Italiano A Sautès-Fridman C . B cells and tertiary lymphoid structures as determinants of tumour immune contexture and clinical outcome. Nat Rev Clin Oncol (2022) 19(7):441–57. doi: 10.1038/s41571-022-00619-z 35365796

[18] Pfannstiel C Strissel PL Chiappinelli KB Sikic D Wach S Wirtz RM . The tumor immune microenvironment drives a prognostic relevance that correlates with bladder cancer subtypes. Cancer Immunol Res (2019) 7(6):923–38. doi: 10.1158/2326-6066.CIR-18-0758 30988029

[19] Zhou L Xu B Liu Y Wang Z . Tertiary lymphoid structure signatures are associated with survival and immunotherapy response in muscle-invasive bladder cancer. Oncoimmunology (2021) 10(1):1915574. doi: 10.1080/2162402X.2021.1915574 34104539 PMC8143239

[20] Balar AV Galsky MD Rosenberg JE Powles T Petrylak DP Bellmunt J . Atezolizumab as first-line treatment in cisplatin-ineligible patients with locally advanced and metastatic urothelial carcinoma: a single-arm, multicentre, phase 2 trial. Lancet (London England) (2017) 389(10064):67–76. doi: 10.1016/S0140-6736(16)32455-2 PMC5568632 27939400

[21] Xia QD Sun JX Xun Y Xiao J Liu CQ Xu JZ . SUMOylation pattern predicts prognosis and indicates tumor microenvironment infiltration characterization in bladder cancer. Front Immunol (2022) 13:864156. doi: 10.3389/fimmu.2022.864156 35418978 PMC8995476

[22] Newman AM Liu CL Green MR Gentles AJ Feng W Xu Y . Robust enumeration of cell subsets from tissue expression profiles. Nat Methods (2015) 12(5):453–7. doi: 10.1038/nmeth.3337 PMC4739640 25822800

[23] Subramanian A Tamayo P Mootha VK Mukherjee S Ebert BL Gillette MA . Gene set enrichment analysis: a knowledge-based approach for interpreting genome-wide expression profiles. Proc Natl Acad Sci U S A (2005) 102(43):15545–50. doi: 10.1073/pnas.0506580102 PMC1239896 16199517

[24] Charoentong P Finotello F Angelova M Mayer C Efremova M Rieder D . Pan-cancer immunogenomic analyses reveal genotype-immunophenotype relationships and predictors of response to checkpoint blockade. Cell Rep (2017) 18(1):248–62. doi: 10.1016/j.celrep.2016.12.019 28052254

[25] Jiang P Gu S Pan D Fu J Sahu A Hu X . Signatures of T cell dysfunction and exclusion predict cancer immunotherapy response. Nat Med (2018) 24(10):1550–8. doi: 10.1038/s41591-018-0136-1 PMC6487502 30127393

[26] Hoshida Y Brunet JP Tamayo P Golub TR Mesirov JP . Subclass mapping: identifying common subtypes in independent disease data sets. PloS One (2007) 2(11):e1195. doi: 10.1371/journal.pone.0001195 18030330 PMC2065909

[27] Maeser D Gruener RF Huang RS . oncoPredict: an r package for predicting in vivo or cancer patient drug response and biomarkers from cell line screening data. Briefings Bioinf (2021) 22(6):bbab260. doi: 10.1093/bib/bbab260 PMC8574972 34260682

[28] Chan TA Yarchoan M Jaffee E Swanton C Quezada SA Stenzinger A . Development of tumor mutation burden as an immunotherapy biomarker: utility for the oncology clinic. Ann Oncol (2019) 30(1):44–56. doi: 10.1093/annonc/mdy495 30395155 PMC6336005

[29] Samstein RM Lee CH Shoushtari AN Hellmann MD Shen R Janjigian YY . Tumor mutational load predicts survival after immunotherapy across multiple cancer types. Nat Genet (2019) 51(2):202–6. doi: 10.1038/s41588-018-0312-8 PMC6365097 30643254

[30] Trüb M Zippelius A . Tertiary lymphoid structures as a predictive biomarker of response to cancer immunotherapies. Front Immunol (2021) 12:674565. doi: 10.3389/fimmu.2021.674565 34054861 PMC8149953

[31] Groeneveld CS Fontugne J Cabel L Bernard-Pierrot I Radvanyi F Allory Y . Tertiary lymphoid structures marker CXCL13 is associated with better survival for patients with advanced-stage bladder cancer treated with immunotherapy. Eur J Cancer (Oxford Engl 1990) (2021) 148:181–9. doi: 10.1016/j.ejca.2021.01.036 33743486

[32] Delvecchio FR Fincham REA Spear S Clear A Roy-Luzarraga M Balkwill FR . Pancreatic cancer chemotherapy is potentiated by induction of tertiary lymphoid structures in mice. Cell Mol Gastroenterol Hepatol (2021) 12(5):1543–65. doi: 10.1016/j.jcmgh.2021.06.023 PMC8529396 34252585

[33] Posch F Silina K Leibl S Mündlein A Moch H Siebenhüner A . Maturation of tertiary lymphoid structures and recurrence of stage II and III colorectal cancer. Oncoimmunology (2018) 7(2):e1378844. doi: 10.1080/2162402X.2017.1378844 29416939 PMC5798199

[34] Garaud S Dieu-Nosjean MC Willard-Gallo K . T Follicular helper and b cell crosstalk in tertiary lymphoid structures and cancer immunotherapy. Nat Commun (2022) 13(1):2259. doi: 10.1038/s41467-022-29753-z 35473931 PMC9043192

[35] Calderaro J Petitprez F Becht E Laurent A Hirsch TZ Rousseau B . Intra-tumoral tertiary lymphoid structures are associated with a low risk of early recurrence of hepatocellular carcinoma. J Hepatol (2019) 70(1):58–65. doi: 10.1016/j.jhep.2018.09.003 30213589

[36] Chaurio RA Anadon CM Lee Costich T Payne KK Biswas S Harro CM . TGF-β-mediated silencing of genomic organizer SATB1 promotes tfh cell differentiation and formation of intra-tumoral tertiary lymphoid structures. Immunity (2022) 55(1):115–28.e9. doi: 10.1016/j.immuni.2021.12.007 35021053 PMC8852221

[37] Liu W Wang W Wang X Xu C Zhang N Di W . Cisplatin-stimulated macrophages promote ovarian cancer migration *via* the CCL20-CCR6 axis. Cancer Letters (2020) 472:59–69. doi: 10.1016/j.canlet.2019.12.024 31866467

[38] Kfoury Y Baryawno N Severe N Mei S Gustafsson K Hirz T . Human prostate cancer bone metastases have an actionable immunosuppressive microenvironment. Cancer Cell (2021) 39(11):1464–78.e8. doi: 10.1016/j.ccell.2021.09.005 34719426 PMC8578470

[39] Fan T Li S Xiao C Tian H Zheng Y Liu Y . CCL20 promotes lung adenocarcinoma progression by driving epithelial-mesenchymal transition. Int J Biol Sci (2022) 18(11):4275–88. doi: 10.7150/ijbs.73275 PMC9295072 35864953

[40] Binnewies M Roberts EW Kersten K Chan V Fearon DF Merad M . Understanding the tumor immune microenvironment (TIME) for effective therapy. Nat Med (2018) 24(5):541–50. doi: 10.1038/s41591-018-0014-x PMC5998822 29686425

[41] Wang F Liu P An H Zhang Y . Sulforaphane suppresses the viability and metastasis, and promotes the apoptosis of bladder cancer cells by inhibiting the expression of FAT−1. Int J Mol Med (2020) 46(3):1085–95. doi: 10.3892/ijmm.2020.4665 PMC7387090 32705150

[42] Zhang Z Yu Y Li P Wang M Jiao W Liang Y . Identification and validation of an immune signature associated with EMT and metabolic reprogramming for predicting prognosis and drug response in bladder cancer. Front Immunol (2022) 13:954616. doi: 10.3389/fimmu.2022.954616 35958586 PMC9359097

[43] The Cancer Genome Atlas Research Network . Comprehensive molecular characterization of urothelial bladder carcinoma. Nature (2014) 507(7492):315–22. doi: 10.1038/nature12965 PMC3962515 24476821

[44] Halstead AM Kapadia CD Bolzenius J Chu CE Schriefer A Wartman LD . Bladder-cancer-associated mutations in RXRA activate peroxisome proliferator-activated receptors to drive urothelial proliferation. eLife (2017) 6:30862. doi: 10.7554/eLife.30862 PMC5720590 29143738

[45] Wing JB Tanaka A Sakaguchi S . Human FOXP3(+) regulatory T cell heterogeneity and function in autoimmunity and cancer. Immunity (2019) 50(2):302–16. doi: 10.1016/j.immuni.2019.01.020 30784578

[46] Gobert M Treilleux I Bendriss-Vermare N Bachelot T Goddard-Leon S Arfi V . Regulatory T cells recruited through CCL22/CCR4 are selectively activated in lymphoid infiltrates surrounding primary breast tumors and lead to an adverse clinical outcome. Cancer Res (2009) 69(5):2000–9. doi: 10.1158/0008-5472.CAN-08-2360 19244125

[47] García-Hernández ML Uribe-Uribe NO Espinosa-González R Kast WM Khader SA Rangel-Moreno J . A unique cellular and molecular microenvironment is present in tertiary lymphoid organs of patients with spontaneous prostate cancer regression. Front Immunol (2017) 8:563. doi: 10.3389/fimmu.2017.00563 28567040 PMC5434117

[48] Schweiger T Berghoff AS Glogner C Glueck O Rajky O Traxler D . Tumor-infiltrating lymphocyte subsets and tertiary lymphoid structures in pulmonary metastases from colorectal cancer. Clin Exp Metastasis (2016) 33(7):727–39. doi: 10.1007/s10585-016-9813-y PMC5035322 27449756

[49] Joshi NS Akama-Garren EH Lu Y Lee DY Chang GP Li A . Regulatory T cells in tumor-associated tertiary lymphoid structures suppress anti-tumor T cell responses. Immunity (2015) 43(3):579–90. doi: 10.1016/j.immuni.2015.08.006 PMC4826619 26341400

[50] Gentles AJ Newman AM Liu CL Bratman SV Feng W Kim D . The prognostic landscape of genes and infiltrating immune cells across human cancers. Nat Med (2015) 21(8):938–45. doi: 10.1038/nm.3909 PMC4852857 26193342

[51] Castino GF Cortese N Capretti G Serio S Di Caro G Mineri R . Spatial distribution of b cells predicts prognosis in human pancreatic adenocarcinoma. Oncoimmunology (2016) 5(4):e1085147. doi: 10.1080/2162402X.2015.1085147 27141376 PMC4839336

[52] Koti M Xu AS Ren KYM Visram K Ren R Berman DM . Tertiary lymphoid structures associate with tumour stage in urothelial bladder cancer. Bladder Cancer (2017) 3(4):259–67. doi: 10.3233/BLC-170120 PMC5676768 29152550

[53] Witjes JA Bruins HM Cathomas R Compérat EM Cowan NC Gakis G . European Association of urology guidelines on muscle-invasive and metastatic bladder cancer: Summary of the 2020 guidelines. Eur urol (2021) 79(1):82–104. doi: 10.1016/j.eururo.2020.03.055 32360052

[54] Morcrette G Hirsch TZ Badour E Pilet J Caruso S Calderaro J . APC germline hepatoblastomas demonstrate cisplatin-induced intratumor tertiary lymphoid structures. Oncoimmunology (2019) 8(6):e1583547. doi: 10.1080/2162402X.2019.1583547 31069152 PMC6492969

[55] Benzerdjeb N Dartigues P Kepenekian V Valmary-Degano S Mery E Avérous G . Tertiary lymphoid structures in epithelioid malignant peritoneal mesothelioma are associated with neoadjuvant chemotherapy, but not with prognosis. Virchows Archiv: an Int J pathol (2021) 479(4):765–72. doi: 10.1007/s00428-021-03099-1 33855595

