# Supplementary figures and images for: Tertiary lymphoid structure patterns aid in identification of tumor microenvironment infiltration and selection of therapeutic agents in bladder cancer
Source: Front Immunol. 2022 Nov 7;13:1049884. doi: 10.3389/fimmu.2022.1049884 (PMC9676505; doi:10.3389/fimmu.2022.1049884)

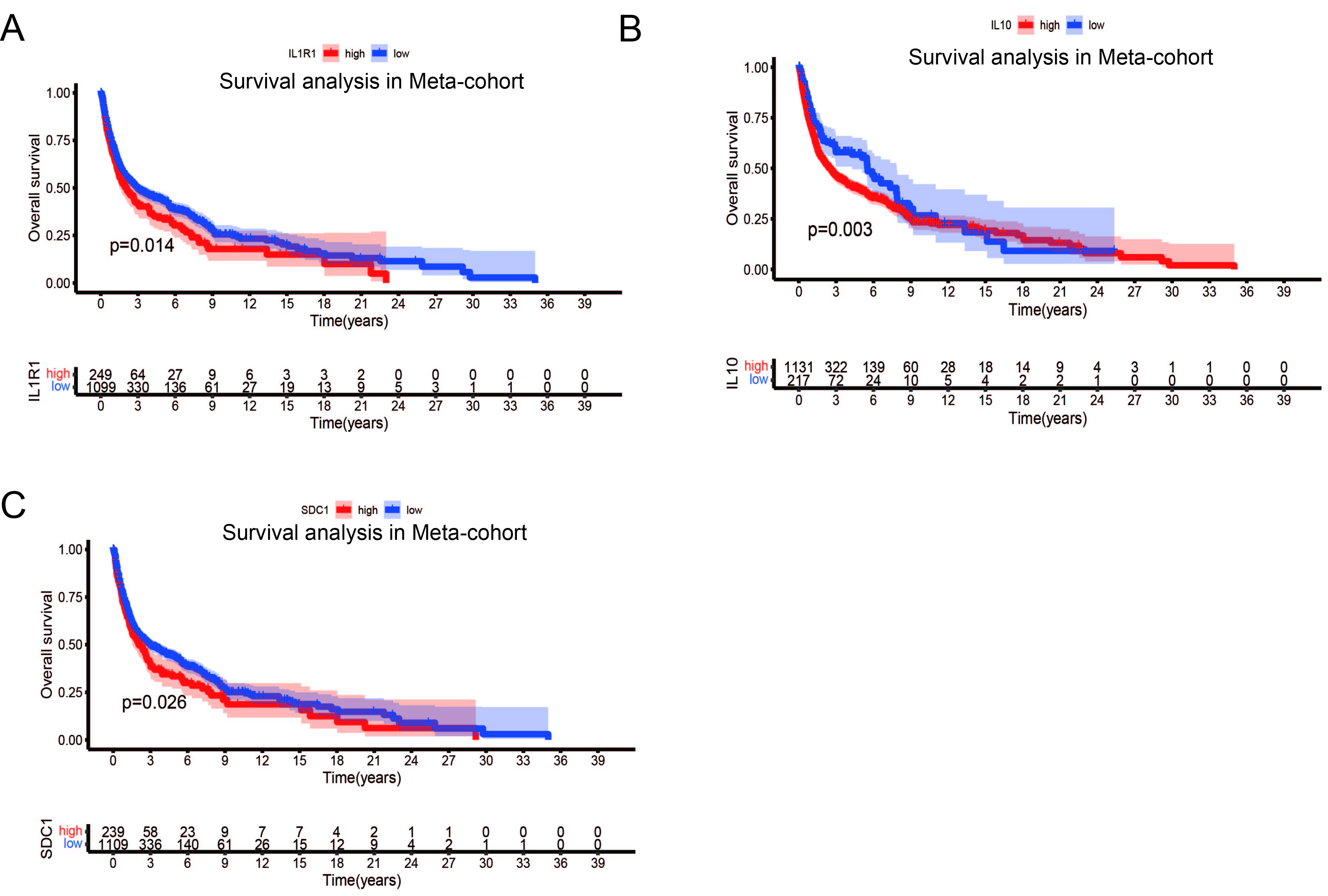

Supplement: Supplementary Figure 1 — Survival analysis for TLS signature genes that low expression subgroup showed survival advantages. (A) Survival analysis for IL1R1. (B) Survival analysis for IL10. (C) Survival analysis for SDC1. [file Image_1.jpeg]

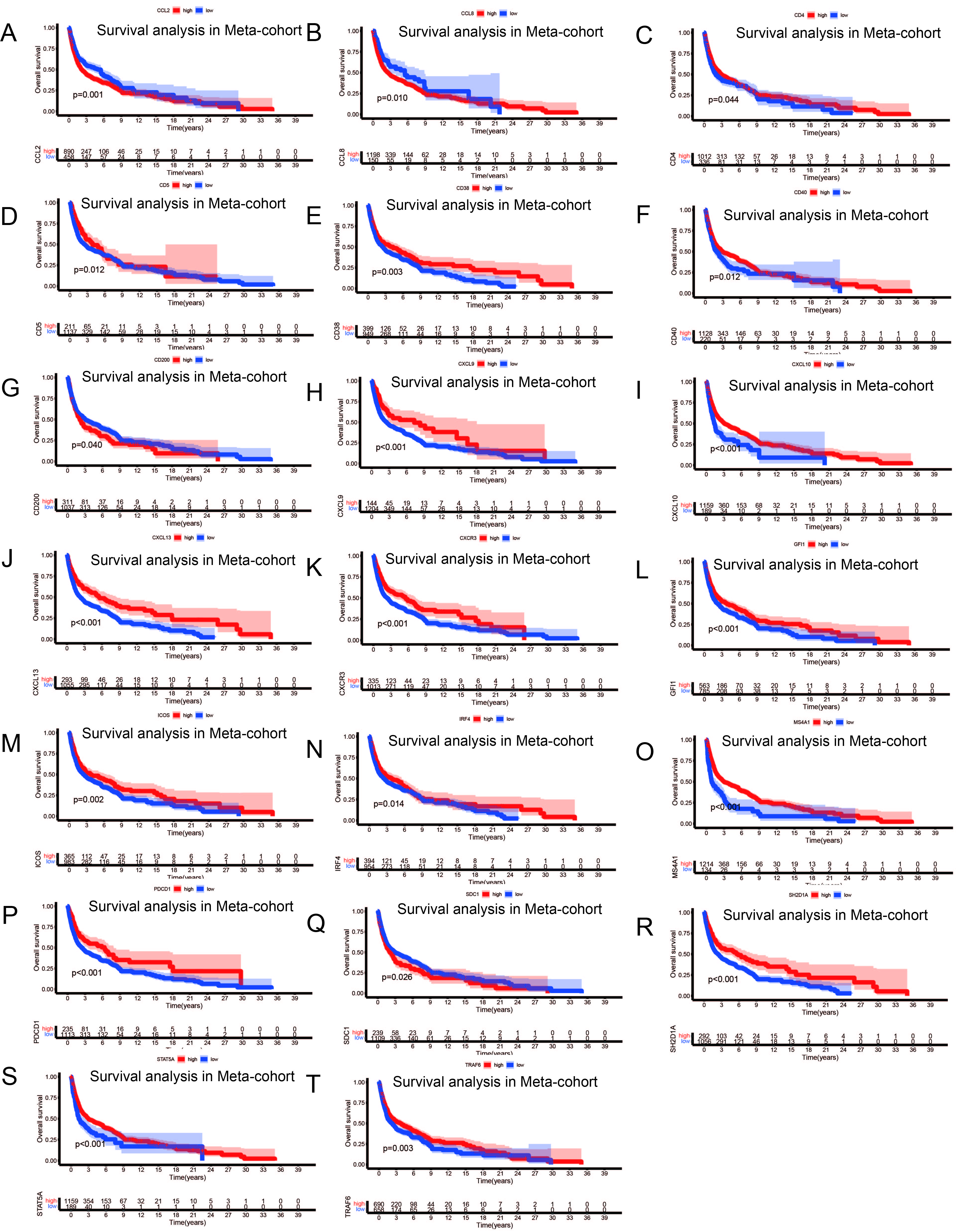

Supplement: Supplementary Figure 2 — Survival analysis for TLS signature genes that high expression subgroup showed survival advantages. (A–S) Survival analysis for CCL2, CCL8, CD4, CD5, CD38, CD40, CD200, CXCL9, CXCL10, CXCL13, CCR3, GFI1, ICOS, IRF4, MS4AA, PDCD1, SH2D1, STAT5 and TRAF6. [file Image_2.jpeg]

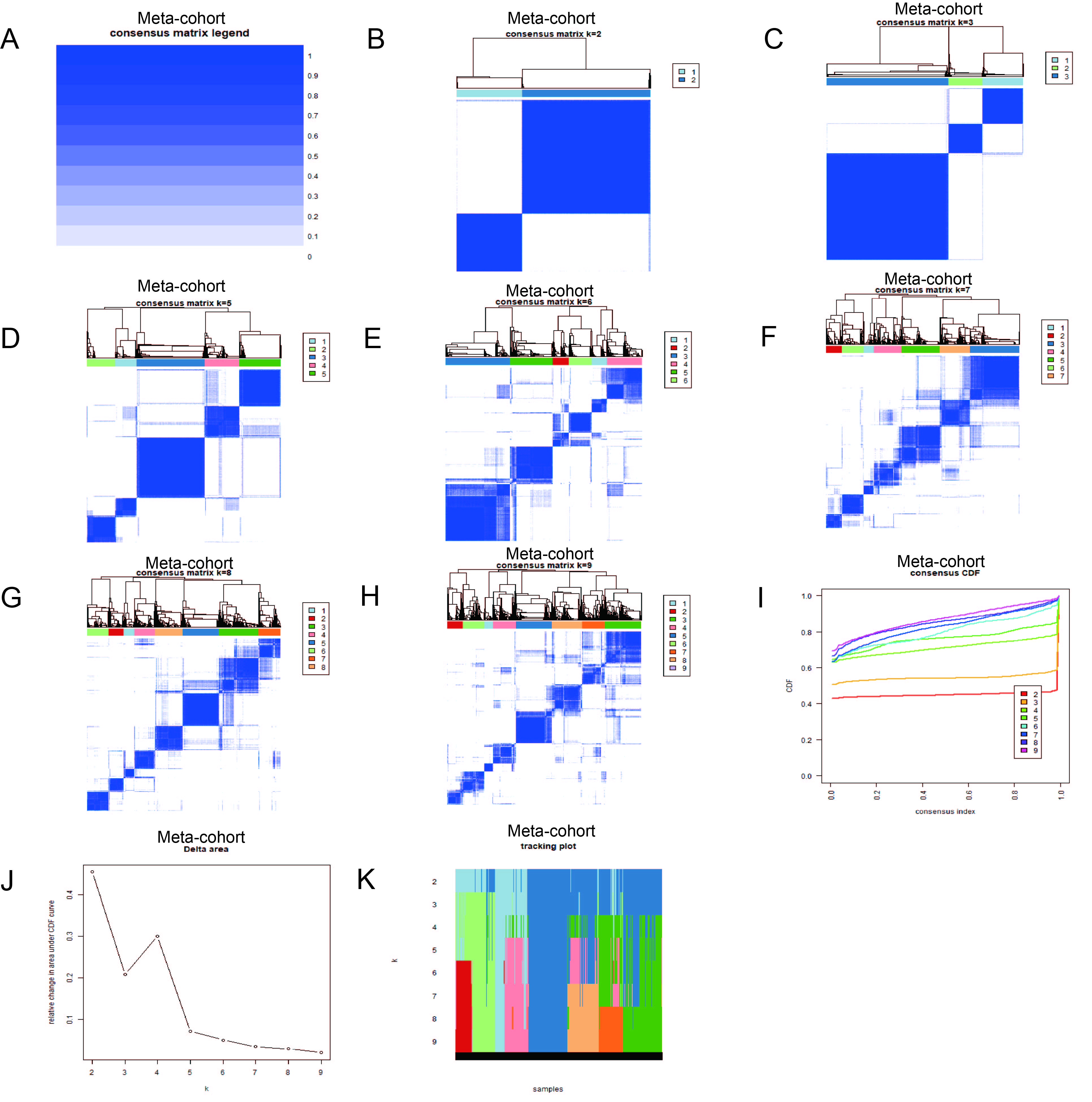

Supplement: Supplementary Figure 3 — The process of generating four distinct TLS cluster related DEGs genomic patterns. (A–H) Unsupervised clustering of 33 TLS cluster related DEGs in meta-cohort and consensus matrices for k = 1, 3 - 9. (I) The cumulative distribution function (CDF) curve for k = 2 - 9. (J) The scree plot for k = 2 - 9. K The track plot for k = 2 - 9. [file Image_3.jpeg]

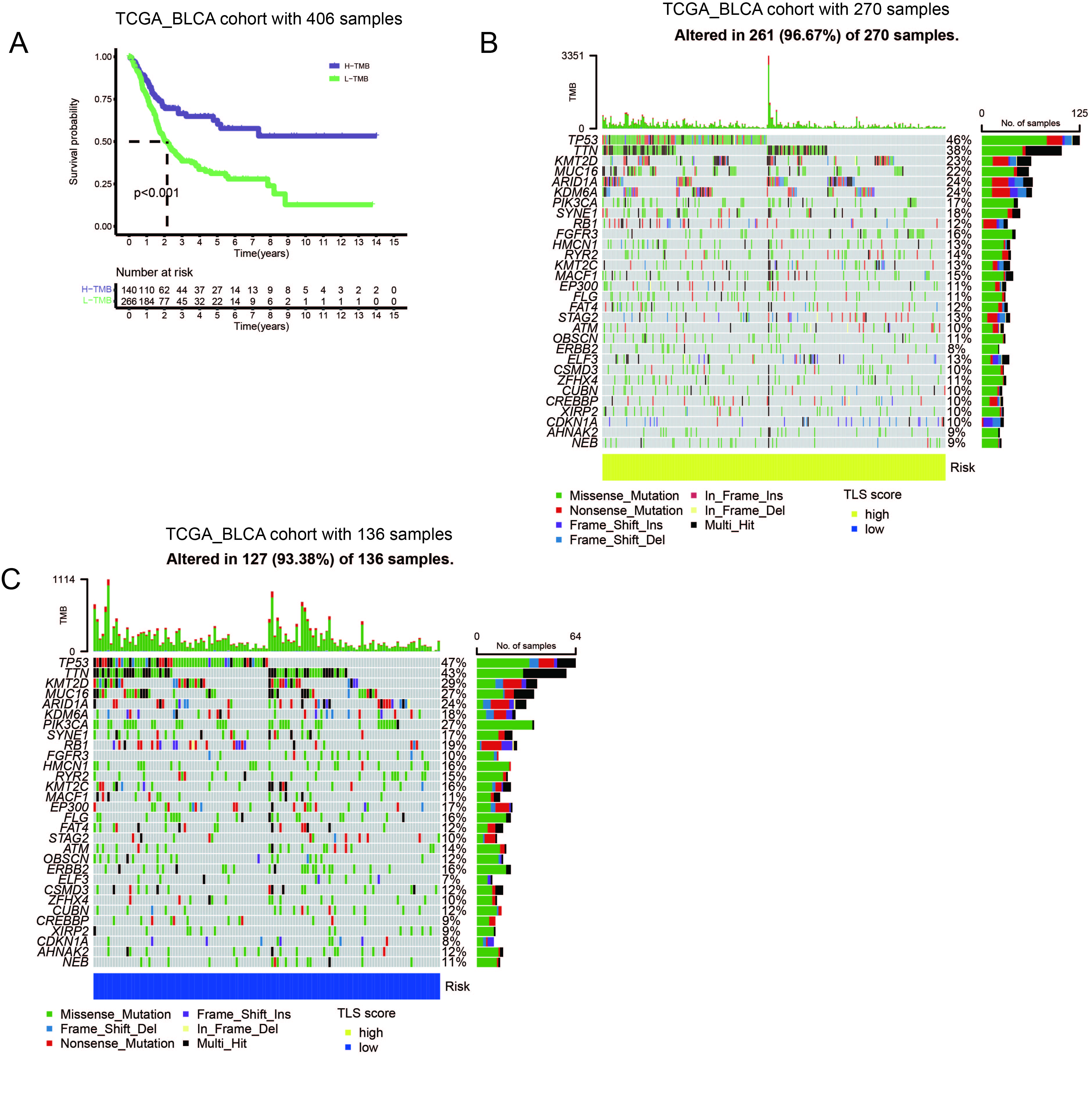

Supplement: Supplementary Figure 4 — Survival analysis between high and low TMB subgroups and somatic mutation between high and low TLS score groups. A Survival analysis between high and low TMB subgroups. B Distribution differences of somatic mutation between high score and low score groups in the TCGA_BLCA cohort. [file Image_4.jpeg]

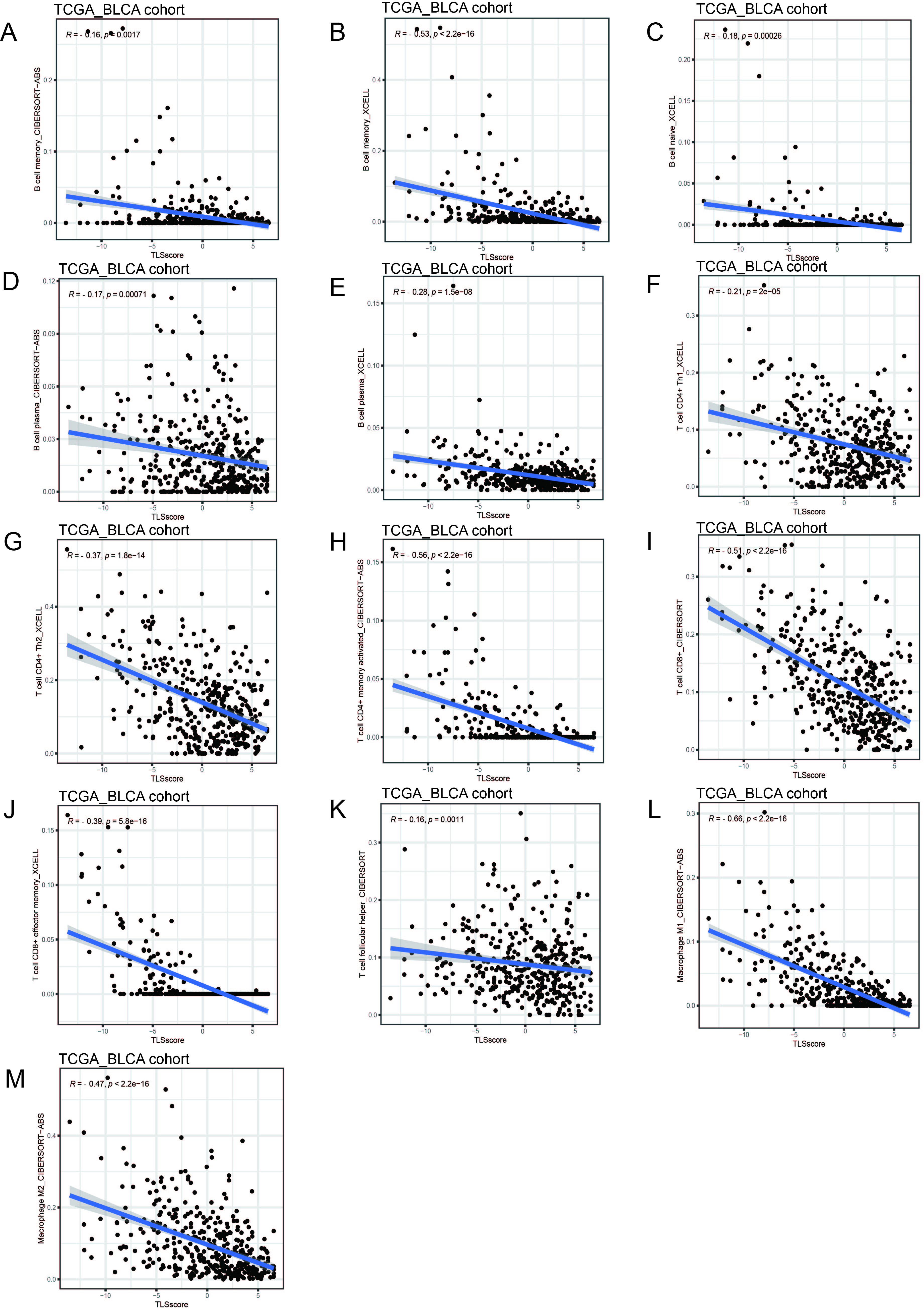

Supplement: Supplementary Figure 5 — Linear regression analysis for TLS score and immune cells. (A) B cell memory. (B) B cell memory. (C) B cell naive. (D) B cell plasma. (E) B cell plasma. (F) T cell CD4+ Th1. (G) T cell CD4+ Th2. (H) T cell CD4+ memory actived. (I) T cell CD8+. (J) T cell CD8+ effector memory. (K) T cell follicular helper. (L) Macrophage M1. (M) Macrophage M2. [file Image_5.jpeg]

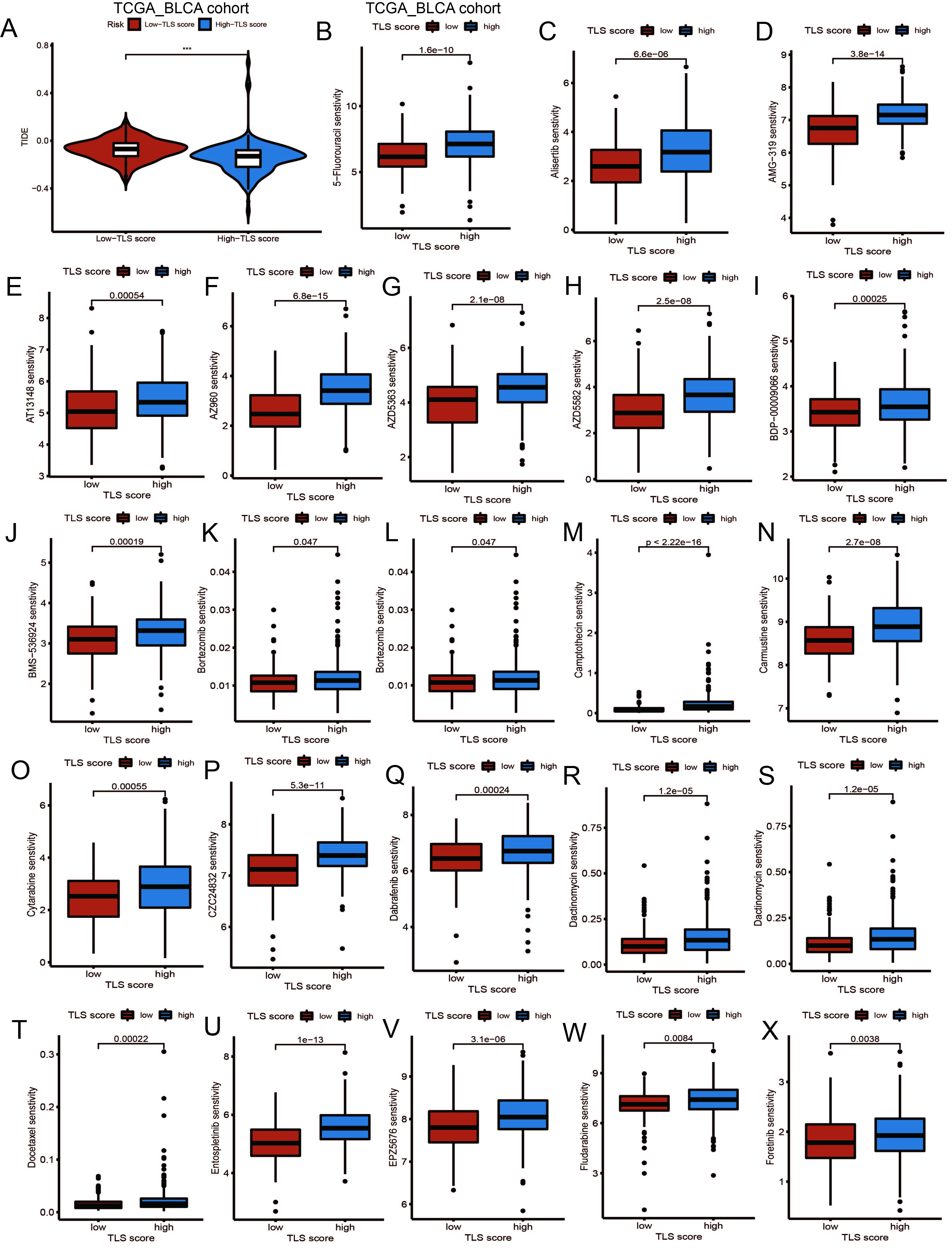

Supplement: Supplementary Figure 6 — 23 drugs that are correlated with TLS score. [file Image_6.jpeg]

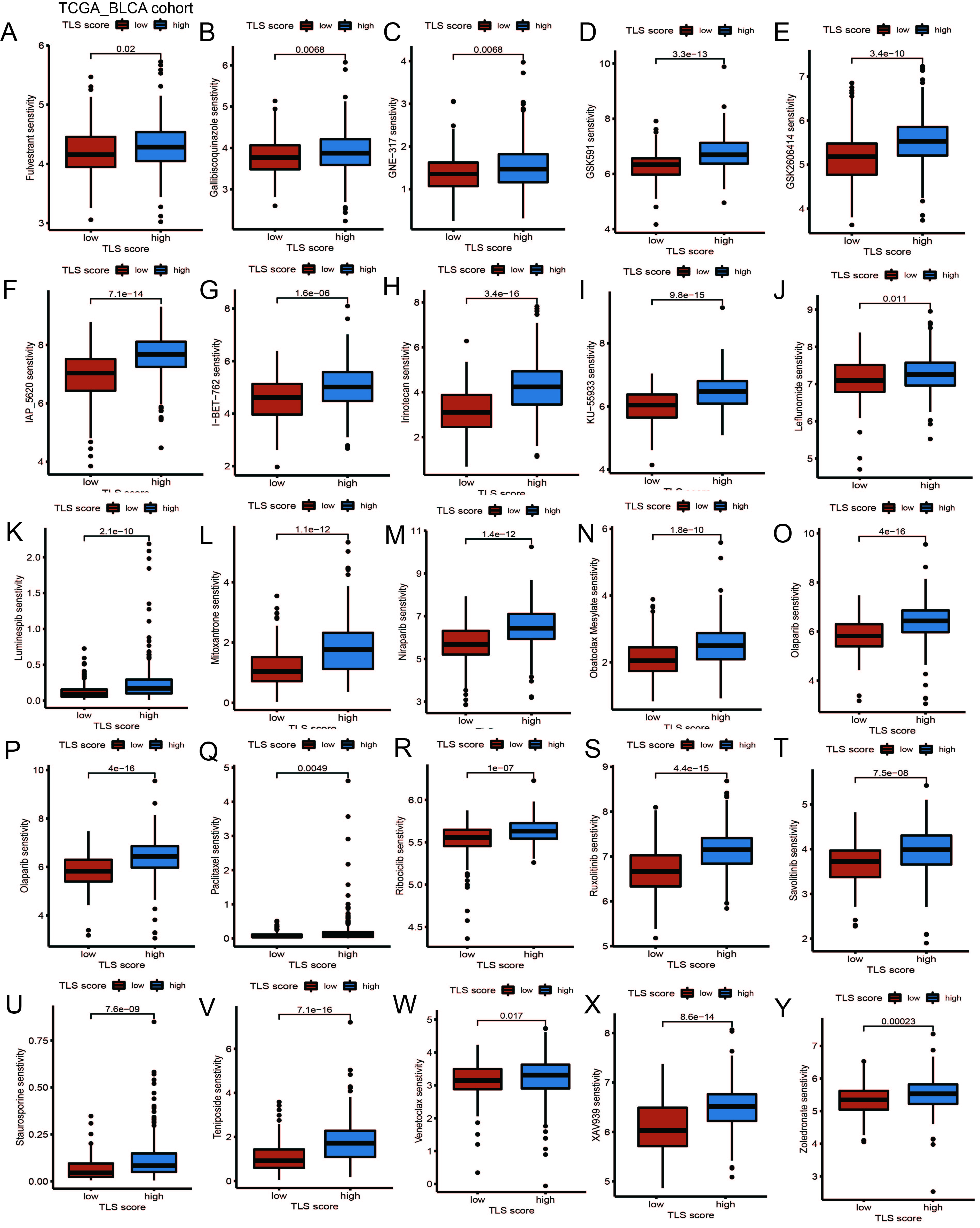

Supplement: Supplementary Figure 7 — 25 drugs that are correlated with TLS score. [file Image_7.jpeg]
